# Comparison of HIV-1 Vif and Vpu accessory proteins for delivery of polyepitope constructs harboring Nef, Gp160 and P24 using various cell penetrating peptides

**DOI:** 10.1371/journal.pone.0223844

**Published:** 2019-10-31

**Authors:** Kimia Kardani, Atieh Hashemi, Azam Bolhassani

**Affiliations:** 1 Department of Pharmaceutical Biotechnology, School of Pharmacy, Shahid Beheshti University of Medical Sciences, Tehran, Iran; 2 Department of Hepatitis and AIDS, Pasteur Institute of Iran, Tehran, Iran; Translational Health Science & Technology Institute, INDIA

## Abstract

To develop an effective therapeutic vaccine against HIV-1, prediction of the most conserved epitopes derived from major proteins using bioinformatics tools is an alternative achievement. The epitope-driven vaccines against variable pathogens represented successful results. Hence, to overcome this hyper-variable virus, we designed the highly conserved and immunodominant peptide epitopes. Two servers were used to predict peptide-MHC-I binding affinity including NetMHCpan4.0 and Syfpeithi servers. The NetMHCIIpan3.2 server was utilized for MHC-II binding affinity. Then, we determined immunogenicity scores and allergenicity by the IEDB immunogenicity predictor and Algpred, respectively. Next, for estimation of toxicity and population coverage, ToxinPred server and IEDB population coverage tool were applied. After that, the MHC-peptide binding was investigated by GalexyPepDock peptide-protein flexible docking server. Finally, two different DNA and peptide constructs containing Nef-Vif-Gp160-P24 and Nef-Vpu-Gp160-P24 were prepared and complexed with four various cell penetrating peptides (CPPs) for delivery into mammalian cells (MPG and HR9 CPPs for DNA delivery, and CyLoP-1 and LDP-NLS CPPs for protein delivery). Our results indicated that the designed DNA and peptide constructs could form non-covalent stable nanoparticles at certain ratios as observed by scanning electron microscope (SEM) and Zetasizer. The flow cytometry results obtained from *in vitro* transfection of the nanoparticles into HEK-293T cell lines showed that the percentage of GFP expressing cells was about 38.38 ± 1.34%, 25.36% ± 0.30, 54.95% ± 0.84, and 25.11% ± 0.36 for MPG/pEGFP-*nef-vif-gp160-p24*, MPG/pEGFP-*nef-vpu-gp160-p24*, HR9/pEGFP-*nef-vif-gp160-p24* and HR9/pEGFP-*nef-vpu-gp160-p24*, respectively. Thus, these data showed that the DNA construct harboring *nef-vif-gp160-p24* multi-epitope gene had higher efficiency than the DNA construct harboring *nef-vpu-gp160-p24* multi-epitope gene to penetrate into the cells. Moreover, delivery of the recombinant Nef-Vif-Gp160-P24 and Nef-Vpu-Gp160-P24 polyepitope peptides in HEK-293T cells was confirmed as a single band about 32 kDa using western blot analysis. Although, both DNA and peptide constructs could be successfully transported by a variety of CPPs into the cells, but the difference between them in transfection rate will influence the levels of immune responses for development of therapeutic vaccines.

## Introduction

Vaccination has been one of the most powerful strategies to reduce global eradication of pathogens and infectious diseases [1, 2]. Due to an urgent need for an effective human immunodeficiency virus (HIV) vaccine, scientists have done too many efforts toward developing an efficient vaccine against HIV-1 in the last decades [3]. Up to now, about six HIV-1 vaccine efficacy trials have been completed. Most these vaccines work through induction of protective antibody responses. Thus, it is required to stimulate effectively both humoral and cellular immune responses in HIV therapeutic vaccines [4]. HIV-1 has four different phylogenetically groups including major (M), outlier (O), non-M-non-O (N), and P. The M group was divided into 9 different subtypes [5]. Table 1 shows the epidemiology of HIV-1 in 2018.

**Table 1:** Epidemiology of HIV-1 infection: UNAIDS data 2018.

Country	New Infections	AIDS-related deaths	People living with HIV
World	1 800 000 [1 400 000–2 400 000]	940 000 [670 000–1 300 000]	36 900 000 [31 100 000–43 900 000]
Iran	4700 [1400–11 000]	3500 [2100–6000]	60 000 [31 000–110 000]
Australia	1000 [800–1100]	<200 [<100– <500]	26 000 [23 000–29 000]
Argentina	6500 [5600–7200]	2000 [1400–2600]	120 000 [110 000–130 000]
Malaysia	7800 [7000–8500]	4400 [3700–5200]	87 000 [76 000–99 000]
Portugal	710 [<500–1600]	<500 [<200– <500]	40 000 [35 000–44 000]
Philippines	12 000 [11 000–13 000]	760 [510–1000]	68 000 [61 000–76 000]
Russia	100 000 [85 000–120 000]	NA	1 000 000 [780 000–1 200 000]
Austria	<200 [<200– <500]	<100 [<100– <100]	7400 [6600–8200]

Compared to traditional vaccines, subunit vaccines showed several benefits including cost-effectiveness, great safety profile, high stability, low toxicity, and ease of manufacturing process. For example, peptide vaccination could be considered as an efficient strategy not only in viral infections, but also in Alzheimer’s disease and even allergic reactions [4, 6]. However, the determination of epitopes or antigens that can enhance efficient preventive or therapeutic immune responses is one of the most important steps in subunit vaccine development [7]. Epitope-based vaccine (EV) is a novel vaccination strategy which has been recently considered by researchers (8); because, the lack of dangerous sequences and infectious parts of virus make this type of vaccine safer than other types. Moreover, both humoral and cellular immune responses can be also effectively stimulated by the selected epitopes [1, 8, 9]. However, EVs have several disadvantages including low stability, delivery issues, heterogeneity of human immunogenetics and low induction of CTL responses [2, 10]. Overcoming the heterogeneity problem, EVs can be designed in a way that the multiple epitopes of viral antigens are covered. Due to hypervariability, this vaccination strategy seems to be really effective against HIV-1 infection [8]. Multi-epitope based vaccines against HIV, Hepatitis B virus (HBV) and human papillomavirus type 16 (HPV16) was successfully developed [8, 11]. Furthermore, the selection of epitopes was one of the most critical steps in EV design. Several databases, predictive methods and software have been developed to correctly choose the immunodominant epitopes [12, 13].

As known, the ~9 kb RNA of HIV-1 contains nine open reading frames for *env*, *gag*, *pol*, *vif*, *vpu*, *nef*, *vpr*, *tat*, and *rev* genes. Env encodes the gp160 polyprotein cleaved to the gp120 (Surface, SU) and gp41 (Transmembrane, TM) subunits which act as virion envelope. Gag is translated as four proteins with structural roles including matrix (MA or p17), capsid (CA or p24), p6, and nucleocapsid (NC or p7). The p24, a 231-residue CA capsid protein, was shown to be probably involved in particle assembly as well as virus entry into a new cell. This protein was revealed to be one of the most highly conserved proteins in HIV-1. Thus, the p24 protein can be considered as a proper candidate for vaccine development. On the other hand, integrase (IN), reverse transcriptase (RT) and protease (PR) are three proteins with enzymatic roles encoded by *pol* gene. The accessory proteins are Vif, Vpu, Nef and Vpr, and the regulatory proteins are Tat and Rev [14, 15]. Vif and Nef can affect assembling and maturation of the virions leading to an increase in viral infectivity [16, 17]. Viral protein U (Vpu) is a 16-kDa phosphorylated integral membrane protein. Several roles have been corresponded to Vpu including CD4 degradation, virion release and ion channel [14, 18].

As mentioned earlier, one of the main disadvantages of EVs is their low ability to induce CTL responses. The use of adjuvants and novel delivery systems seems to be necessary to elicit strong CTL responses in this vaccination strategy [2, 7, 19]. Cell penetrating peptides (CPPs) were shown to be effective in protection and entry of macromolecules such as proteins, nucleic acids, peptide nucleic acids, liposomes and inorganic particles [20, 21]. Moreover, these safe and non-toxic agents could transfer cargoes with the size of 200 nm in diameter and the concentration of 100 mM through the cell membrane [21]. Among hundred types of CPPs, MPG and HR9 peptides were demonstrated to have great potential as gene delivery systems. The MPG is an amphipathic peptide that forms stable non-covalent nanoparticles with DNA [20, 22, 23]. The MPG contains different domains such as SV40 nuclear localization sequence or NLS (PKKRKV), a spacer (WSQP), and N-terminal hydrophobic domain derived from HIV glycoprotein 41 (gp41) [20, 23, 24]. HR9, an arginine-rich peptide, can form non-covalently complexes with nanoparticles and deliver them via direct membrane translocation pathway [22, 25]. Ponnappan and colleagues identified two novel CPPs including LDP-NLS and CyLoP-1. In LDP-NLS, LDP (Latarcin-derived peptide) derived from spider toxin Latarcin 1 (Lt1) was conjugated to NLS derived from SV40 antigen. CyLoP-1 is a cysteine-rich peptide. Previously, their efficiency was successfully proved in HeLa cells for protein delivery [26, 27].

Our current study was undertaken to choose and analyze the most conserved and immunogenic epitopes of HIV-1 Nef, Vif, Vpu, Gp160 and P24 proteins. Two polyepitope constructs including Nef-Vif-Gp160-P24 and Nef-Vpu-Gp160-P24 were designed using computational and bioinformatics tools. Then, the expression of the gene constructs (*nef-vif-gp160-p24* and *nef-vpu-gp160-p24*) was performed in *E*.*coli* expression system for production of the recombinant polyepitope peptides (rNef-Vif-Gp160-P24 and rNef-Vpu-Gp160-P24). Finally, the efficiency of DNA and peptide delivery was studied using four types of CPPs *in vitro*.

## Materials and methods

### Protein sequence retrieval

The reference protein sequence of HIV-1 main group (M group) including Nef, Vif, Vpu, envelope glycoprotein 160 (Gp160), and P24 were retrieved in FASTA format from HIV sequence database (https://www.hiv.lanl.gov/content/sequence/HIV/mainpage.html).

### Prediction of T-cell epitopes

In order to design multiepitope peptide constructs using immunoinformatic analysis, at first, we determined potentially immune-protective epitopes. T cells identify antigens as short peptide segment in association with major histocompatibility complex (MHC) molecules on antigen-presenting cells [6]. There are two categories of T-cells: a) CD8^+^ T cytotoxic cells which recognize peptides displayed by MHC-I molecules, b) CD4^+^ T helper cells which recognize epitopes related to MHC-II molecules. As opposed to B-cell epitopes, T-cell epitopes only recognize linear peptides. MHC-I binding prediction was very strong and had wide allelic coverage by integration with predictions of proteasomal cleavage and TAP binding sites. MHC-II binding prediction was not as advanced as MHC-I binding prediction, *i*.*e*., it is still developing at a fast rate [6].

#### Prediction of MHC-I binding profile for the conserved epitopes

T cell epitopes were predicted by tools available in NetMHCpan 4.0 and Syfpeithi, which provide a catalog of experimentally characterized T cell epitopes as well as data on MHC binding and MHC ligand elution experiments.

#### NetMHCpan-4.0

NetMHCpan-4.0 server predicts binding of peptides to the known MHC molecules using ANNs method. NetMHCpan-4.0 is a method trained on binding affinity. Predictions were possible for peptides of length 8–41 and for 41 animals including monkey, mouse *etc*. with ~ 75–80% accuracy for peptide binding to HLA class I molecules. Furthermore, the user can perform a prediction against any custom MHC class I molecule by uploading its full-length sequence [28]. In this study, threshold (percentile rank) was set at 0.5% for strong binders and 2% for weak binders (default thresholds, S1 Table).

#### SYFPEITHI

SYFPEITHI database (http://www.syfpeithi.de/0-Home.htm) was used to predict peptide binding to MHC class I molecule using a support vector machine (SVM) multitask Kernel-based [28]. SYFPEITHI (http://www.syfpeithi.de/0-Home.htm) is a database of confirmed peptide sequences of human, mouse, and other organisms, recognized as intrinsic binders of MHC class I and MHC class II molecules [29].

#### Tap transport/ proteasomal cleavage

Prediction of proteasomal cleavage sites is an alternative way to predict T cell epitopes, but prediction sensitivity is not very high, since proteasomal cleavage is just the first step in epitope generation process and during later stages, the majority of peptides are non-MHC binders. TAP transport is an inconsequential step in epitope generation process in terms of epitope prediction, since many TAP binders are not naturally presented by MHC molecules [13]. In current study, NetCTL 1.2 server combined with Tap transport/proteasomal cleavage tools (http://www.cbs.dtu.dk/services/NetCTL/) was used to predict antigen processing through the MHC class I antigen presentation pathway. In this method, weight on C-terminal cleavage set on 0.15, Tap transport efficiency fixed as 0.05 and epitope identification settled on 0.75 (default values) [28].

#### Prediction of MHC-II binding profile for the conserved epitopes

NetMHCIIpan 3.2 server (http://www.cbs.dtu.dk/services/NetMHCIIpan/) was used to predict the binding of peptides and MHC class II. Analysis of all protein sequences was performed individually. Hence, threshold (percentile rank) for strong and weak binders was set at 2% and 10% (default threshold, S1 Table), respectively.

### Allergenicity assessment

Due to the importance of proteins in inducing allergenic reaction, the ability to predict the potential allergenicity has become crucial especially in the field of genetically modified foods, therapeutics, bio-pharmaceuticals *etc* [28]. The allergenicity of the epitopes was analyzed by http://crdd.osdd.net/raghava/algpred/submission.html server. The SVM module based on amino acid composition was employed as default method.

### Immunogenicity analysis

An essential factor to clarify the difference between epitope and non-epitope peptides is epitope immunogenicity [29]. In this study, we evaluated immunogenicity scores by the IEDB immunogenicity predictor (http://tools.iedb.org/immunogenicity/). To determine the immunogenicity scores, default parameters were applied.

### Toxicity analysis

To investigate toxicity of the selected epitopes, the web server ToxinPred (http://crdd.osdd.net/raghava/toxinpred/) was used. This website provided the confirmation of non-toxicity of epitopes for the host according to all physicochemical parameters. ToxinPred was used with default parameters.

### Population coverage calculation

To ensure the universal coverage within a heterogeneous population, it is crucial to calculate global population coverage for the chosen epitopes since the HLAs are among the most polymorphic proteins and vary among different geographical regions around the world [6]. The epitopes have a different binding profile with various HLA alleles; thus, population coverage must be taken into a different set of alleles to cover all regions and to get desirable immune response in all individuals within a given population. For that reason, all promising MHC-I and MHC-II epitope candidates were assessed for population coverage against different geographic areas through IEDB population coverage calculation tool at http://tools.iedb.org/population/ [6].

### Molecular docking analysis

**Fig 1:**
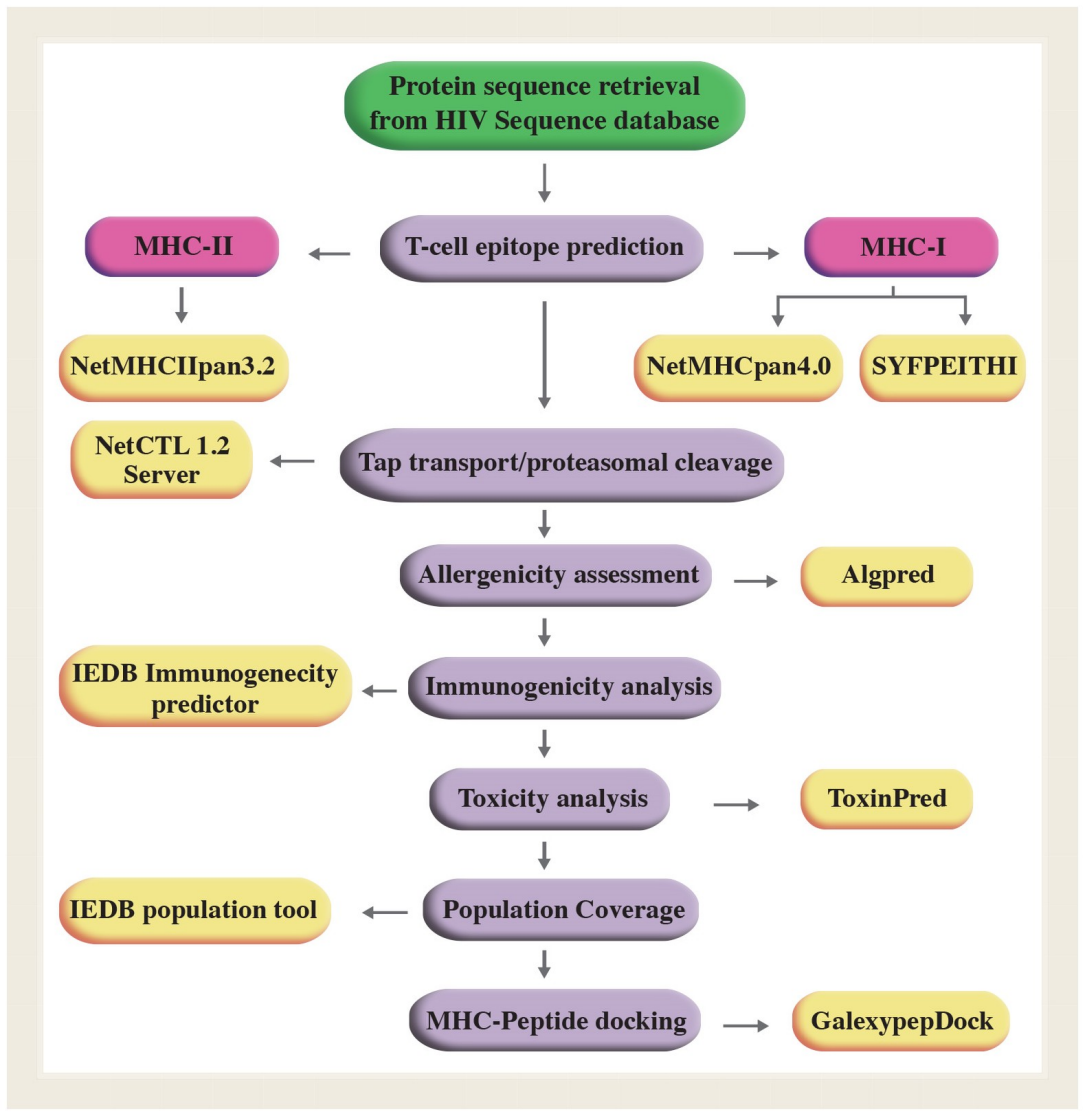
The overall process of polyepitope peptides design.

To evaluate the formation of MHC-peptide complex, we used GalexyPepDock peptide-protein flexible docking server (http://galaxy.seoklab.org/cgibin/submit.cgi?type=PEPDOCK). Among computational approaches, molecular docking is one of the most frequently used methods for *in silico* prediction of the structure of peptide-protein complexes. Fig 1 shows the design of polyepitope peptide constructs in general. To prevent the creation of junctional neo-epitopes and optimize the cleavage of proteasome, the flexible linker (AAY) was incorporated between epitopes. The methionine residue (M) was added to the N-terminal domain to begin the translation, and the six histidine residues (H) were added to the C-terminal domain for purification and detection of the final product using the anti-His antibody *in vitro*.

### *In vitro* analysis

**Fig 2:**
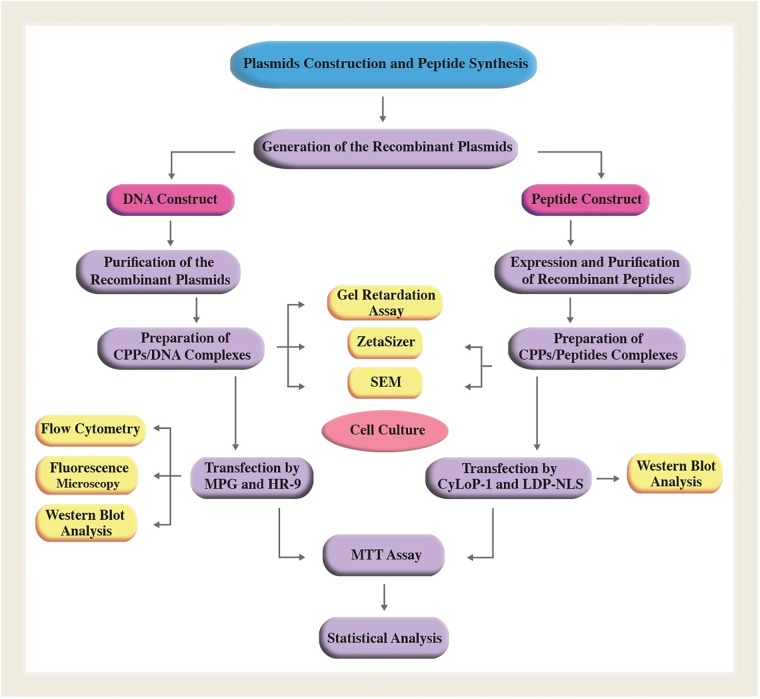
The overall process of *in vitro* studies.

The overall process of *in vitro* analysis was indicated in Fig 2.

#### Plasmids construction and peptide synthesis

**Fig 3:**
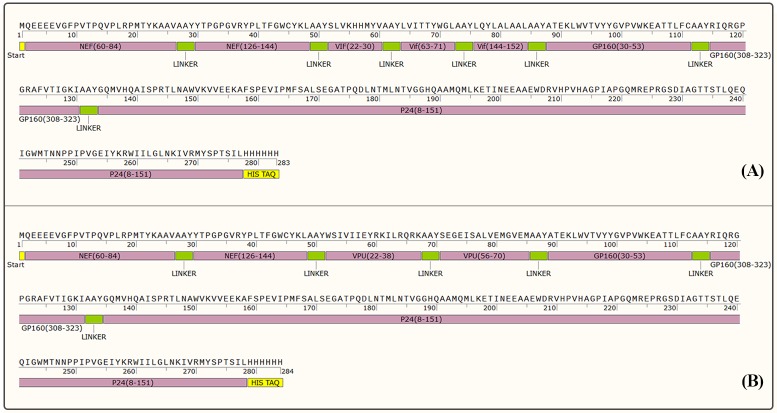
The design of polyepitope peptide constructs. (A) The Nef-Vif-Gp160-P24 polyepitope construct; (B) The Nef-Vpu-Gp160-P24 polyepitope construct.

The pUC57 cloning vector containing the HIV-1 selected epitopes as fusion with AAY linker (pUC-*nef-vif-gp160-p24* and pUC-*nef-vpu-gp160-p24*) was prepared by Biomatik Corporation (Cambridge, Canada) as shown in Fig 3. It should be mentioned that the nucleotide sequence of *nef-vif-gp160-p24* and *nef-vpu-gp160-p24* was obtained by amino acid reverse translation tool (http://www.bioinformatics.org/sms2/rev_trans.html). Moreover, the MPG, HR9, LDP-NLS and CyLoP-1 peptides were synthesized from Biomatik Company. The sequences and charges of cell penetrating peptides were shown in Table 2.

**Table 2:** The properties of cell penetrating peptides.

CPP	Sequence	Length	Net charge	MW(g/mol)
MPG	ALFLGFLGAAGSTMGAWSQPKKKRKV	26	+5	2750.27
HR-9	CHHHHHRRRRRRRRRHHHHHC	21	+9.9	3001.38
LDP-NLS	KWRRKLKKLRPKKKRKV	17	+12	2276.91
CyLoP-1	CRWRWKCCKK	10	+4.8	1396.76

#### Preparation of DNA constructs

**Fig 4:**
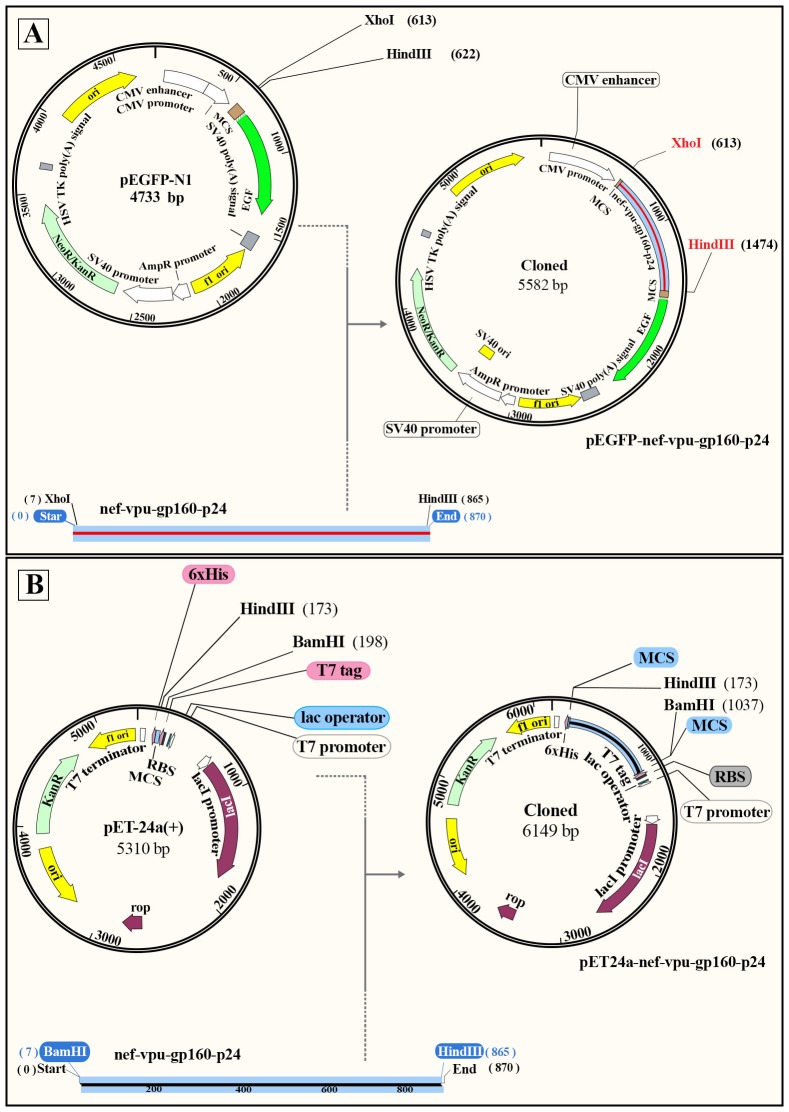
The schematic process of cloning. A) The *nef-vpu-gp160-p24* fragment was digested from pUC57 with *Xho*I and *Hind*III, and then was ligated into pEGFP-N1 vector; B) The *nef-vpu-gp160-p24* fragment was digested from pUC57 with *Bam*HI and *Hind*III and then ligated into pET24a (+) vector.

The pEGFP-N1 (Clontech) was considered as a mammalian expression vector. In order to generate pEGFP-N1-*nef-vif-gp160-p24* and pEGFP-N1-*nef-vpu-gp160-p24*, the *nef-vif-gp160-p24* and *nef-vpu-gp160-p24* genes were subcloned from pUC57-*nef-vif-gp160-p24* and pUC57-*nef-vpu-gp160-p24* into the *Xho*I and *Hind*III cloning sites of pEGFP-N1 expression vector, respectively as shown in Fig 4A. The recombinant plasmids were purified by ion exchange chromatography with DNA extraction mini kit (Yekta Tajhiz Azma, Iran). DNA concentration and purity were assessed via spectrophotometric determination at 260 nm using NanoDrop spectrophotometer. The presence of the inserted fragments was confirmed by digestion with the restriction enzymes as detected on gel electrophoresis.

#### Preparation of the CPPs/DNA complexes

The synthetic HR9 and MPG peptides were dissolved in water (Conc. 2 mg/ml) as a stock solution. 2 μg of plasmid DNA (pEGFP-*nef-vif-gp160-p24* or pEGFP-*nef-vpu-gp160-p24*) was mixed with HR9 and MPG in PBS1X (pH = 7.4) at different N/P ratios, and incubated for 1 hour (h) at room temperature. The N/P ratios were used as 0.5, 1, 2, 5, 10 and 2, 5, 10 for HR9/DNA and MPG/DNA for both constructs, respectively. The association between CPPs and DNA was assessed by gel retardation assay. Moreover, to determine the stability of HR9/DNA and MPG/DNA complexes against DNA nucleases, DNase I was added to the complexes with a final concentration of 1.37 U/ μg DNA, and the mixtures were incubated at 37°C for 1 h followed by the addition of stop solution (200 mM sodium chloride, 20 mM EDTA and 1% SDS) [30]. To assess the serum stability, the nanoparticles at the N/P ratios of 5:1 and 10:1 for HR9/DNA, and MPG/DNA, respectively, were exposed to 10% serum and incubated for 5 h at 37°C. Then, DNA plasmids were released from protein by adding 10% SDS solution for 2 h, and analyzed by electrophoresis on agarose gel 1% [31]. Then, the zeta potential of the HR9/DNA and MPG/DNA complexes was determined using a dynamic light scattering detector (Zetasizer, Nano-ZS, Malvern, UK). Moreover, the morphology and size of HR9/DNA and MPG/DNA nanoparticles was obtained by scanning electron microscope (SEM) at the N/P ratios of 5:1 and 10:1, respectively (KYKY-EM3200 model, China).

#### Cloning and expression of the recombinant polyepitope peptides

The *nef-vif-gp160-p24* and *nef-vpu-gp160-p24* genes from pUC57 vectors were inserted into *Bam*HI and *Hind*III restriction sites of the pET24a (+) vector, a T7 promoter based plasmid (Invitrogen, USA) as shown in Fig 4B. The *E*. *coli* strains of BL21 (DE3) and Rosetta (DE3) were used as expression hosts. These hosts were transformed with the expression plasmids harboring the genes of interest. The transformants were selected on Luria-Bertani (LB) agar plate and inoculated into 3 ml of Luria-Bertani (LB) broth containing 100 μg/ ml kanamycin (Sigma). The culture was shaken at 37°C overnight, and then transferred to Ty2x medium. Once the cell density (OD) at wavelength of 600 nm reached 0.7–0.8, the expression was induced by adding 1 mM IPTG (SinaClon bioscience Co, Iran), and the induced culture was shaken for 1, 2, 3, 4 and 16 h under the same conditions. The cell pellets were collected and analyzed by SDS-PAGE in a gel containing 12% (W/V) polyacrylamide (SDS gel apparatus; BioRad), followed by staining with coomassie brilliant blue. Also, the expression was confirmed by western blotting using anti-His antibody (Abcam, USA).

#### Purification of the recombinant polyepitope peptides under denaturing conditions

The recombinant polyepitope peptides (rNef-Vif-Gp160-P24 and rNef-Vpu-Gp160-P24) were purified under denaturing conditions by affinity chromatography method on a nickel-nitrilotriacetic acid (Ni-NTA)-agarose column (Qiagen, Germany) using 6xHis-tag according to the manufacturer procedure. Finally, the purified protein fractions were dialyzed against phosphate-buffer saline 1X (PBS1X; Dialysis membrane, MWCO: 3500 kDa). The concentration of proteins was measured by Bradford protein assay kit and NanoDrop spectrophotometer at 280 nm and stored at -70 °C.

#### Preparation of the CyLoP-1/peptide and LDP-NLS/peptide complexes

To form the CyLoP-1/rNef-Vif-Gp160-P24, LDP-NLS/rNef-Vif-Gp160-P24, CyLoP-1/rNef-Vpu-Gp160-P24 and LDP-NLS/rNef-Vpu-Gp160-P24 complexes, the CyLoP-1 and LDP-NLS CPPs were mixed with an equal amount (1 μg) of the recombinant polyepitope peptides at molar ratio of 10:1 (CPP: peptide, [26, 27]), and incubated for 60 min at room temperature. Then, the size and morphology of the complexes were determined by SEM. Moreover, the charge of nanoparticles was assessed by Zetasizer Nano ZS (Malvern Instruments, UK) at 25°C.

#### Cell culture

Human embryonic kidney (HEK-293T; ATCC: CRL-3216^™^) cells were obtained from cell bank at the Pasteur Institute of Iran and cultured in RPMI 1640 medium (Sigma, Germany) supplemented with 10% heat inactivated fetal bovine serum (FBS, Gibco, Germany), penicillin (100 U/ml) and streptomycin (0.1 mg/ml) under standardized conditions (95% relative humidity, 5% CO_2_, 37°C).

#### DNA delivery by MPG and HR9 CPPs *in vitro*

In order to investigate MPG and HR9 mediated delivery of pEGFP-*nef-vif-gp160-p24* and pEGFP-*nef-vpu-gp160-p24*, HEK-293T cells were grown in a RPMI 1640 medium with 10% FBS. Then, HEK-293T cells were seeded into 24-well plates at the density of 5×10^4^ cells per well. Once reached ~85% confluency, the MPG/pEGFP-*nef-vif-gp160-p24* and MPG/pEGFP-*nef-vpu-gp160-p24* nanoparticles were added to the medium in the presence of 5% serum. After 6 h incubation at 37°C, the culture medium was replaced with the complete RPMI 5% FBS. Moreover, at 85% confluency, the HR9/pEGFP-*nef-vif-gp160-p24* and the HR9/pEGFP-*nef-vpu-gp160-p24* nanoparticles were gently added to the cells in serum-free medium. After 1h incubation at 37°C, the culture medium was replaced with the complete RPMI 10% FBS. The TurboFect/pEGFP-*nef-vif-gp160-p24* and TurboFect/pEGFP-*nef-vpu-gp160-p24* complexes were used as a positive control (Fermentas, Germany) according to the manufacturer’s instructions. The expression of constructs (transfection efficiency) was monitored by fluorescence microscopy (Envert- Fluorescent Ceti, Korea) as well as flow cytometry analysis (Partec, Germany) at 48 h post-transfection.

#### *In vitro* delivery of the polyepitope peptides by LDP-NLS and CyLoP-1 CPPs

For CyLop-1 and LDP-NLS-mediated delivery of the recombinant polyepitope peptides, the CyLoP-1/rNef-Vif-Gp160-P24, LDP-NLS/rNef-Vif-Gp160-P24, CyLoP-1/rNef-Vpu-Gp160-P24 and LDP-NLS/ rNef-Vpu-Gp160-P24 nanoparticles were formed at a molar ratio of 10:1, and incubated for 1h at room temperature. The cells were grown in a RPMI 1640 medium with 10% FBS. Then, HEK-293T cells were seeded into 24-well plates at the density of 5×10^4^ cells per well. Once reached ∼85% confluency, cells were overlaid with these nanoparticles. After 2h incubation at 37°C, the cells were treated with trypsin-EDTA and washed with PBS1X. The TurboFect/rNef-Vif-Gp160-P24 and TurboFect/rNef-Vpu-Gp160-P24 complexes were used as a positive control (Pro-Ject^™^ Reagent, Fermentas, Germany) according to the manufacturer’s instructions. The delivery of rNef-Vif-Gp160-p24 and rNef-Vpu-Gp160-P24 was confirmed by western blot analysis using anti-His antibody (Abcam, USA).

#### Cytotoxicity of the nanoparticles

The cytotoxicity of MPG/pEGFP-*nef-vif-gp160-p24* (N:P = 10:1), HR9/pEGFP-*nef-vif-gp160-p24* (N/P = 5:1), CyLoP-1/rNef-Vif-Gp160-P24 (molar ratio of CPP:peptide = 10:1), LDP-NLS/rNef-Vif-Gp160-P24 (molar ratio of CPP:peptide = 10:1), MPG/pEGFP-*nef-vpu-gp160-p24* (molar ratio of CPP:peptide = 10:1), HR9/pEGFP-*nef-vpu-gp160-p24* (molar ratio of CPP:peptide = 10:1), CyLoP-1/rNef-Vpu-Gp160-P24 (molar ratio of CPP:peptide = 10:1), and LDP-NLS/rNef-Vpu-Gp160-P24 (molar ratio of CPP:peptide = 10:1) nanoparticles were investigated in HEK-293T cell line. At first, the cells were grown in RPMI 1640 medium with 10% FBS. Then, HEK-293T cells were seeded into 96-well plates at the density of 1×10^4^ cells per well. Once reached ∼85% confluency, they were incubated with certain doses of the CPPs/ DNA nanoparticles as well as the CPPs/polyepitope peptide complexes for 48 h. Finally, the cytotoxicity was evaluated in a colorimetric assay using 3-(4, 5- dimethylthiazol-2-yl)-2, 5-diphenyltetrazolium bromide (MTT). Indeed, the cells were treated with 100 μl of MTT solution (0.5 mg/well, Sigma, Germany), and incubated further for 3 h at 37°C in humidified CO_2_. The formazan crystals were then solubilized with 100 μl dimethyl sulfoxide (DMSO). Absorbance was measured at 570 nm using an ELISA reader (Labsystems Multiskan MS 352 Microplate Reader). The percentage of viable cells was calculated according to the following equations:

$$\begin{array}{c} Cell\;viability\;\left(\%\right)\;=\;\left(Absorbance\;of\;test/Absorbance\;of\;control\right)\;\times \;100\\ Cytotoxicity\;\left(\%\right)\;=\;100-\%\;cell\;viability \end{array}$$

#### Statistical analysis

Student’s *t*-test was performed to analyze the transfection percentage of the nanoparticles using flow cytometry and their cytotoxicity. The value of *p* < 0.05 was considered statistically significant.

## Results

### *In silico* analysis

#### T-cell epitope prediction of MHC-I

At initial step, the selected peptide sequences of HIV-1 virus including Nef, Vif, Vpu, Gp160, and P24 were analyzed by Syfpeithi and NetMHCpan 4.0 servers to determine the highly putative immunodominant regions. As known, the binding of epitopes to MHC-I molecules is the most selective step for antigen presentation to CTLs. Table 3 indicates the selected CTL epitopes based on binding affinity. In this study, we have selected the best epitopes with the strongest binding affinity to interact with MHC class I alleles. NEF_60–71_, NEF_72–84_, VIF_63–71_, VPU_22–30_, VPU_29–37_, VPU_56–64_, VPU_61–70_, Gp160_41−53,_ and P24_8−151_ epitopes have the strongest binding affinity.

**Table 3:** T-cell epitope prediction of HIV-1.

Epitopes	Position	Syfpeithi Average Scores	NetMHCpan Average Rank Scores	Prediction Score	Proteasomal C -terminal cleavage Score	Tap Transport Efficiency
VIF _LVITTYWGL_	63–71	22	1.9753	0.9836	0.9357	1.1640
VPU _WSIVIIEYR_	22–30	21	0.2318	0.1464	0.3443	1.5650
VPU _YRKILRQRK_	29–37	23	1.5265	1.5467	0.8926	0.6470
VPU SEGEISALV	56–64	21	0.0699	1.0496	0.9454	0.0670
VPU SALVEMGVE	61–70	15	2.0134	0.1231	0.0500	-1.4440
NEF QEEEEVGFPVTP	60–71	25	0.0163	1.3218	0.8485	-0.2180
NEF QVPLRPMTYKAAV	72–84	27	0.0033	1.7099	0.9066	0.3670
Gp160 GVPVWKEATTLFC	41–53	18	0.0110	1.6856	0.9669	2.6960

#### T-cell epitope prediction of MHC-II

MHC-II restricted CD4^+^ T-cells activation is important to induce and maintain an efficient antibody or CTL response. In this study, we used NetMHCIIpan server for predictive analysis. Based on the fact that a good T-cell epitope should interact with multiple HLA alleles, top scoring epitopes with the highest number of binding HLADR alleles were selected as putative helper T-cell epitope candidates. Table 4 indicates the selected helper T cell epitopes of HIV-1 based on binding affinity. Two epitopes of NEF_126–144,_ and Gp160_308−323_ demonstrated higher binding affinity to interact with MHC class II alleles than other epitopes. Neither of these two epitopes were allergen nor toxin.

**Table 4:** T-cell epitope prediction of MHC-II.

Epitopes	Position	NetMHCIIpan Average Rank Scores	Toxicity	Allergenicity	Immunogenicity score
NEF YTPGPGVRYPLTFGWCYKL	126–144	46	Non-Toxin	Non-Allergen	0.33873
Gp160 RIQRGPGRAFVTIGKI	308–323	19	Non-Toxin	Non-Allergen	0.37806

#### Proteasomal cleavage, TAP transport efficiency, immunogenicity and allergenicity

The MHC-I presents, and T cell screens peptides originated from intracellular proteins. The proteasome is the place of proteasomal cleavage and prepare proteolytic segments for antigen presenting [32]. After presentation, some peptides from the cytosol are transferred to the endoplasmic reticulum (ER) through TAP. Peptides with 8–12 residues long and the accurate binding motif would load onto MHC class I. After that, the Golgi transfers the MHC-I/peptide complex towards the cell surface. Finally, T cells recognize these MHC-I ligands. Thus, it is necessary to predict the MHC-I ligands for vaccine design [29, 32]. In order to predict proteasomal cleavage and TAP transport efficiency scores, the epitopes were investigated by NetCTL 1.2 Server tool. The results indicated that he NEF_60–71_, NEF_72–84_, VIF_63–71_, VPU_22–30_, VPU_29–37_, VPU_56–64_, VPU _61–70_, and Gp160_41−53_ epitopes had the highest prediction scores (1.3218, 1.7099, 0.9836, 0.1464, 1.5467, 1.0496, 0.1231 and 1.6856, respectively) that indicates the great efficiency of proteasomal cleavage and tap transport. Also, the immunogenicity scores of the selected epitopes were determined through the IEDB immunogenicity predictor. As observed in Table 5, the selected epitopes were not allergen or toxin.

**Table 5:** Allergenicity, immunogenicity and toxicity of the selected epitopes.

Epitopes	Position	Allergenicity	Immunogenicity Score	Toxicity
VIF _LVITTYWGL_	63–71	Non-Allergen	0.32332	Non-Toxin
VPU _WSIVIIEYR_	22–30	Non-Allergen	0.42196	Non-Toxin
VPU _YRKILRQRK_	29–37	Non-Allergen	0.03432	Non-Toxin
VPU SEGEISALV	56–64	Non-Allergen	0.11216	Non-Toxin
VPU SALVEMGVE	61–70	Non-Allergen	0.02286	Non-Toxin
NEF QEEEEVGFPVTP	60–71	Non-Allergen	0.46503	Non-Toxin
NEF QVPLRPMTYKAAV	72–84	Non-Allergen	-0.25908	Non-Toxin
Gp160 GVPVWKEATTLFC	41–53	Non-Allergen	0.31262	Non-Toxin

#### Population coverage analysis

The population coverage results have been indicated in Table 6. Eight epitopes including VIF_63–71_, VPU_22–30_, VPU_29–37_, VPU_56–64_, VPU_61–70_, NEF_60–71_, NEF_72–84,_ and Gp160_41−53_ had interaction with the most frequent MHC class I alleles, and examined population coverage against the worldwide (89.24%, 63.42%, 33.56%, 68.15%, 61.18%, 32.36%, 63.88% and 58.84%, respectively). The epitope Gp160_308−323_ determined population coverage against worldwide 52.34% in interaction with MHC class II alleles. Moreover, the epitope NEF_126–144_ has interaction with both MHC class I and MHC class II alleles. Also, the population coverage of the selected epitopes was investigated in different countries that have high rate of HIV-1 prevalence (Table 6).

**Table 6:** Population coverage using IEDB population coverage tool.

Epitopes Country	VIF_63–71_	VPU_22–30_	VPU_29–37_	VPU_56–64_	VPU_61–70_	NEF_60–71_	NEF_72–84_	NEF_126–144_	Gp160_41−53_	Gp160_308−323_
World	87.86%	63.42%	33.56%	68.15%	61.18%	32.36%	63.88%	37.61%	58.84%	52.34%
Iran	79.47%	63.36%	46.68%	55.23%	68.53%	53.49%	78.83%	9.46%	48.04%	31.66%
Australia	85.53%	53.4%	30.44%	56.56%	50.11%	14.41%	63.58%	45.47%	59.57%	13.9%
Argentina	73.9%	62.14%	31.06%	66.34%	65.35%	13.89%	69.74%	11.81%	54.2%	28.01%
Malaysia	75.89%	56.13%	35.6%	45.35%	59.7%	15.16%	36.43%	19.66%	44.93%	20.35%
Portugal	91.59%	63.85%	35.55%	78.2%	64.3%	38.28%	66.46%	31.47%	46.76%	50.7%
Philippines	93.85%	60.31%	18.62%	68.64%	60.31%	1.99%	34.54%	53.76%	46.23%	2.41%
Russia	88.48%	52.7%	32.05%	63.48%	52.02%	35.34%	67.23%	49.63%	65.07%	42.95%
Austria	92.77%	67.11%	27.9%	80.36%	61.38%	45.54%	59.51%	44.44%	57.53%	69.75%

#### Molecular docking

**Fig 5:**
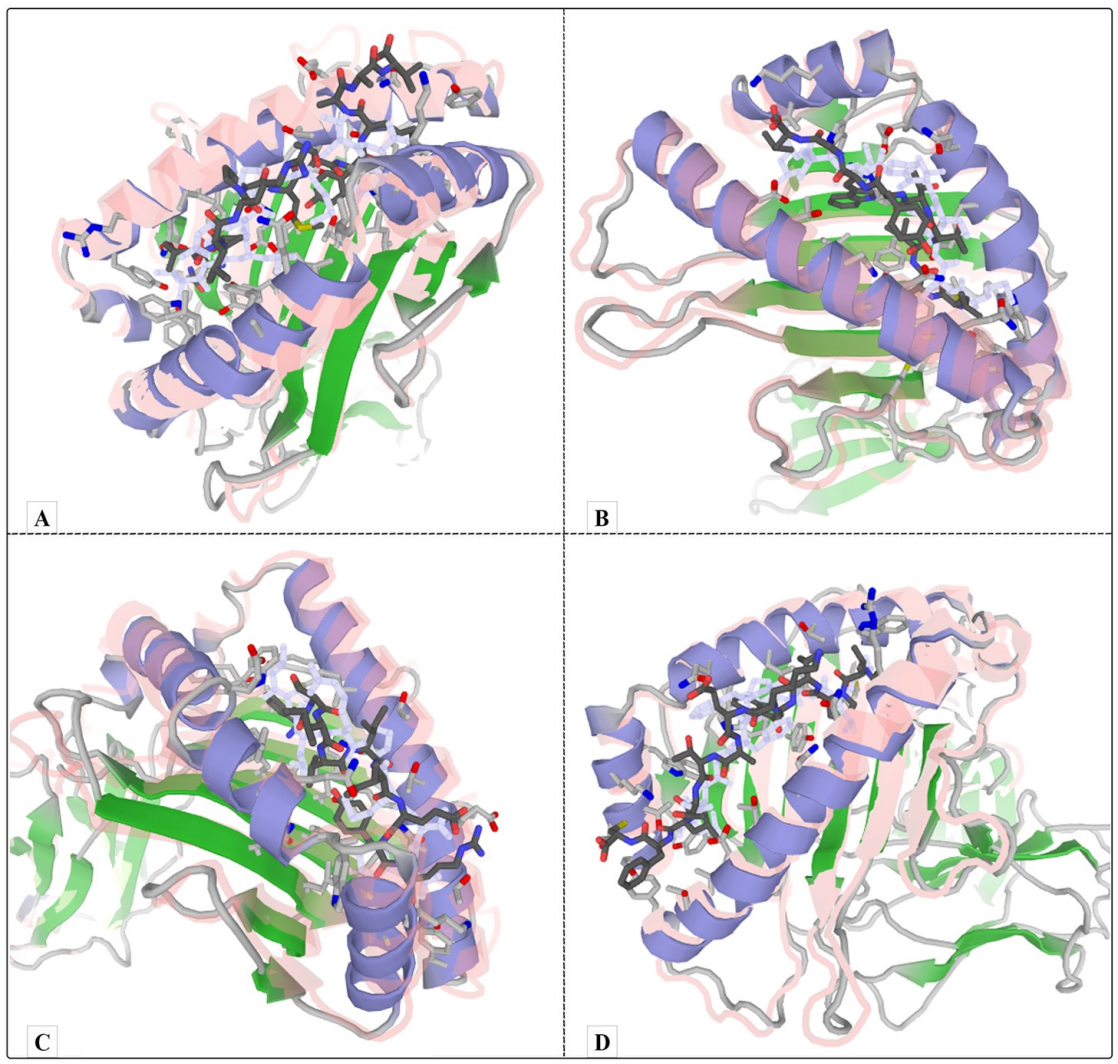
Molecular docking. A) Successful Peptide-Protein Docking between NEF_72–84_ and HLA B5601 with interaction scores of 316.0, B) Successful Peptide-Protein Docking between VIF_63–71_ and HLA A0301 with interaction scores of 216.0, C) Successful Peptide-Protein Docking between VPU_22–30_ and HLA B1301 with interaction scores of 193.0, D) Successful Peptide-Protein Docking between GP160_41−53_ and HLA A6801 with interaction scores of 240.0.

To determine the peptide-protein interaction, available pdb files were obtained from RCSB PDB server (https://www.rcsb.org/). Then, all epitopes and MHC pdb files were applied to server. After the completion of the analysis, Top models with the highest interaction similarity score were selected for each peptide and its MHC (Table 7). Fig 5 indicates an example of successful peptide-protein docking between peptide and MHC protein.

**Table 7:** Peptide-protein interaction similarity scores.

	VIF_63–71_	VPU_22–30_	VPU_29–37_	VPU_56–64_	VPU_61–70_	NEF_60–71_	NEF_72–84_	Gp160_41−53_
**HLA A0301**	216.0	175.0	147.0	151.0	160.0	175.0	279.0	229.0
**HLA A1101**	201.0	175.0	146.0	153.0	173.0	185.0	282.0	228.0
**HLA A2402**	224.0	161.0	161.0	205.0	168.0	187.0	241.0	242.0
**HLA A3401**	199.0	180.0	148.0	153.0	168.0	181.0	281.0	226.0
**HLA A6801**	201.0	176.0	154.0	162.0	169.0	178.0	282.0	240.0
**HLA A7401**	222.0	179.0	153.0	161.0	161.0	181.0	285.0	249.0
**HLA B1301**	209.0	193.0	145.0	173.0	164.0	179.0	309.0	236.0
**HLA B1801**	196.0	190.0	140.0	149.0	152.0	173.0	306.0	232.0
**HLA B3901**	200.0	185.0	141.0	153.0	151.0	178.0	299.0	210.0
**HLA B4101**	209.0	182.0	160.0	163.0	160.0	170.0	298.0	239.0
**HLA B5601**	208.0	198.0	154.0	162.0	169.0	169.0	316.0	235.0
**HLA B5801**	206.0	196.0	143.0	155.0	160.0	171.0	304.0	231.0
**HLA B7301**	205.0	173.0	159.0	160.0	142.0	185.0	289.0	240.0

### *In vitro* studies

#### Confirmation of the DNA constructs

The *nef-vif-gp160-p24* and *nef-vpu-gp160-p24* genes were correctly subcloned into pEGFP-N1 and pET24a vectors. The electrophoresis results showed clear bands of ~867 and ~870 bp related to *nef-vif-gp160-p24* and *nef-vpu-gp160-p24* genes, respectively after digestion with the restriction enzymes.

#### Expression and purification of the recombinant polyepitope peptides

**Fig 6:**
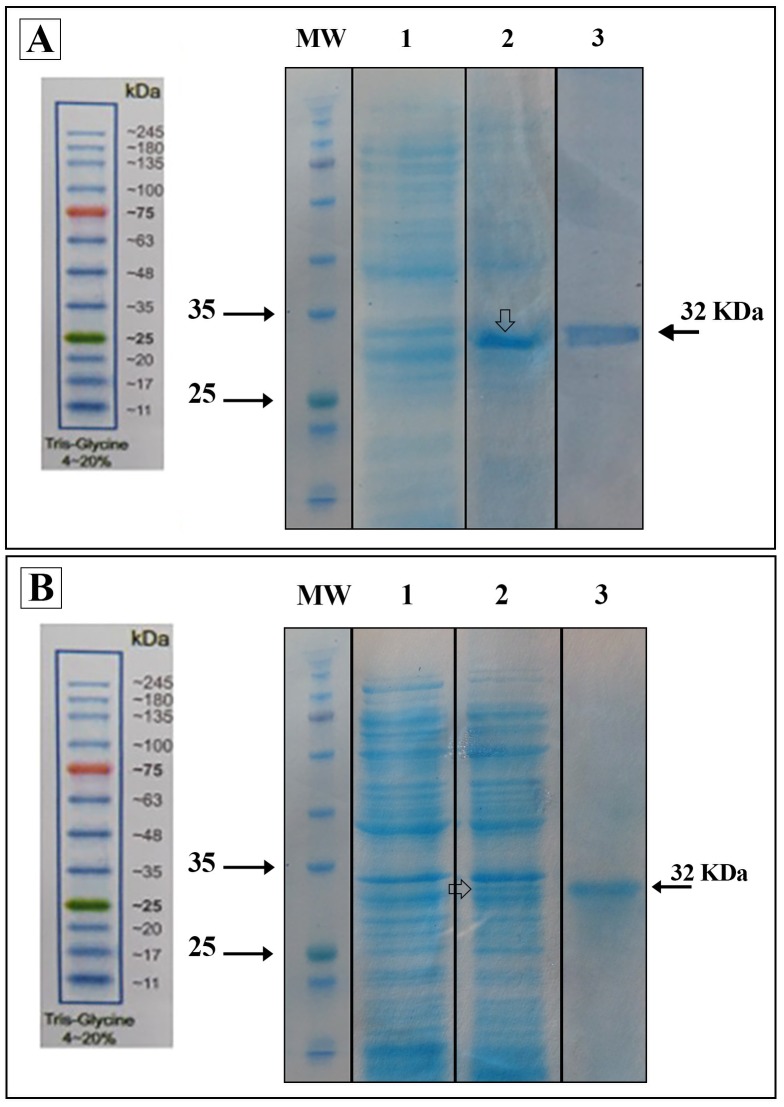
Expression and purification of the recombinant polyepitope peptides in *E*. *coli* Rosetta expression system. (A) Lane 1: Before IPTG induction (BI), Lane 2: After IPTG induction (AI), Lane 3: The purified Nef-Vif-Gp160-P24 polyepitope peptide by affinity chromatography under denaturing conditions. The purified Nef-Vif-Gp160-P24 migrated as a clear band of ~32 kDa in SDS-PAGE; (B) Lane 1: Before IPTG induction (BI), Lane 2: After IPTG induction (AI), Lane 3: The purified Nef-Vpu-Gp160-P24 polyepitope peptide by affinity chromatography under denaturing conditions. The purified Nef-Vpu-Gp160-P24 migrated as a dominant band of ∼32 kDa in SDS-PAGE.

The expression of rNef-Vif-Gp160-P24 and rNef-Vpu-Gp160-P24 polyepitope peptides was evaluated in two bacterial systems including pET-24a/*Rosetta* and pET-24a/*BL21*. Analysis of bacterial lysates via SDS-PAGE after overnight cultivation for Nef-Vif-Gp160-P24 and 4h cultivation for Nef-Vpu-Gp160-P24 in the presence of 1 mM IPTG at 37°C resulted in detection of protein bands at the theoretically expected molecular weight about 32 kDa for both polyepitope peptides in pET-24a/*Rosetta* expression system (Fig 6A and 6B). Moreover, the recombinant polyepitope peptides could be successfully purified under denaturing conditions. The purified polyepitope peptides were detectable as a clear band of 32 kDa using anti-His antibody in western blot analysis.

#### Formation and physicochemical characterization of the CPPs/DNA and CPPs/peptide complexes

**Fig 7:**
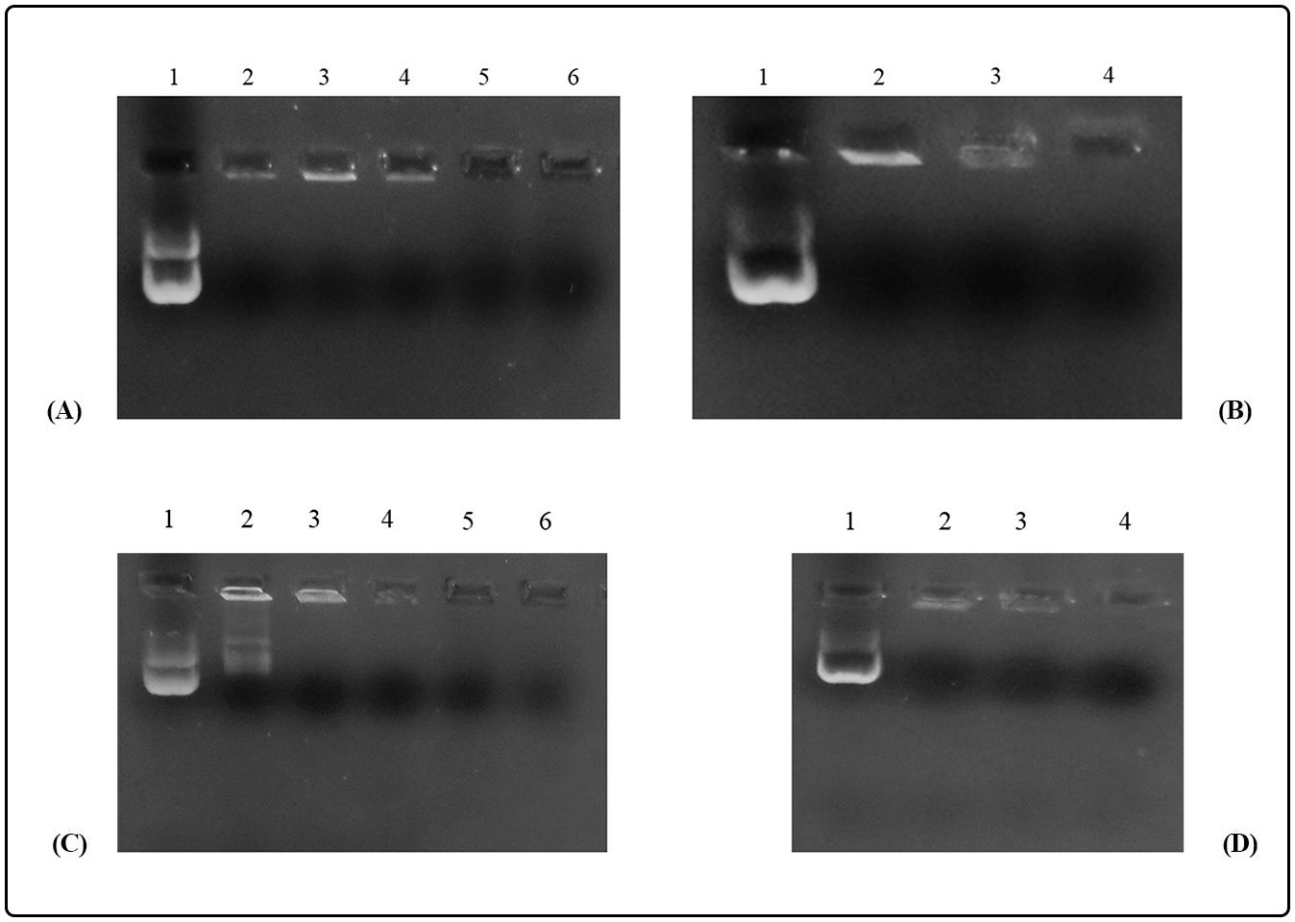
Analysis of the CPPs/DNA complexes. (A) Gel retardation assay of the HR9/pEGFP-*nef-vif-gp160-p24* nanoparticles, Lane 1: pEGFP-*nef-vif-gp160-p24* as a control in the absence of HR9, Lanes 2–6: The HR9/pEGFP-*nef-vif-gp160-p24* complexes at different N/P ratios of 0.5, 1, 2, 5, 10, respectively; (B) Gel retardation assay of the MPG/pEGFP-*nef-vif-gp160-p24* nanoparticles, Lane 1: pEGFP-*nef-vpu-gp160-p24* as a control in the absence of MPG, Lanes 2–4: The MPG/pEGFP-*nef-vif-gp160-p24* complexes at different N/P ratios of 2, 5, 10, respectively; (C) Gel retardation assay of the HR9/pEGFP-*nef-vpu-gp160-p24* nanoparticles, Lane 1: pEGFP-*nef-vpu-gp160-p24* as a control in the absence of HR9, Lanes 2–6: The HR9/pEGFP-*nef-vpu-gp160-p24* complexes at different N/P ratios of 0.5, 1, 2, 5, 10, respectively; (D) Gel retardation assay of the MPG/pEGFP-*nef-vpu-gp160-p24* nanoparticles, Lane 1: pEGFP-*nef-vpu-gp160-p24* as a control in the absence of MPG, Lanes 2–4: The MPG/pEGFP-*nef-vpu-gp160-p24* complexes at different molar ratios of 2, 5, 10, respectively.

The negatively charged pEGFP-*nef-vif-gp160-p24* and pEGFP-*nef-vpu-gp160-p24* interacted with the cationic MPG and HR9 CPPs for generation of the nanoparticles. Indeed, the DNA molecule did not migrate into agarose gel electrophoresis at an N/P ratio of 10:1 for MPG/DNA and 5:1 for HR9/DNA indicating the formation of MPG/pEGFP-*nef-vif-gp160-p24*, MPG/pEGFP-*nef-vpu-gp160-p24*, HR9/pEGFP-*nef-vif-gp160-p24* and HR9/pEGFP-*nef-vpu-gp160-p24* nanoparticles as shown in Fig 7. For stability assay, after DNase I treatment, the naked DNA was degraded, while the HR9/DNA and MPG/DNA complexes protected the DNA from DNase I degradation at the N/P ratios more than 5:1 and 10:1, respectively. For serum protection assay, the same N/P ratios of 5:1 and 10:1 were selected for the HR9/DNA and MPG/DNA complexes, respectively. Our data showed that recovered DNA from nanoparticles remained intact on agarose gel in the presence of serum after 5 h incubation with FBS. In contrast, unprotected plasmid DNA was degraded (S1 Fig). Finally, the zeta potential and size of pEGFP-*nef-vif-gp160-p24*, pEGFP-*nef-vpu-gp160-p24*, rNef-Vif-Gp160-P24, rNef-Vpu-Gp160-P24 proteins, as well as the complexes at certain ratios were measured as shown in Table 8. The SEM results showed that before the complex preparation, the DNA constructs and polyepitope peptides showed amorphous bodies in micrometer size, but after that, all CPPs/DNA and CPPs/polyepitope peptide complexes were recognized as a distinct particle with an average size of 100–250 nm, and 100–150, respectively. In addition, the zeta potential of the complexes was studied by Zetasizer at the same ratios. The DNA and peptides had negative charges, while the CPP/DNA and CPP/peptide complexes showed positive charges that can be considered as an important factor for transferring the complexes through the cell membrane. Of course, the zeta potential of the CyLop-1/peptide complexes showed a negative charge; but however, their small size and more positivity versus each peptide alone can lead to penetrate into the cells.

**Table 8:** Zeta potential and size of the nanoparticles.

Nanoparticle	Zeta potential (mV) Nef-vpu-p24-gp160	Zeta potential (mV) Nef-vif-p24-gp160	Size (nm) Nef-vpu-p24-gp160	Size (nm) Nef-vif-p24-gp160
DNA alone	-13.4	-17.7	3547	2928
MPG/DNA	27.9	21.3	169.9	229.8
HR-9/DNA	28.5	38.4	198.9	119
Protein alone	-9.03	-22.7	4687	5560
CyLoP-1/polyepitope peptide	-1.60	-9.22	119.5	159.6
LDP-NLS/polyepitope peptide	0.411	3.31	140.3	115.8

#### The MPG- and HR9-mediated DNA delivery into mammalian cells

**Fig 8:**
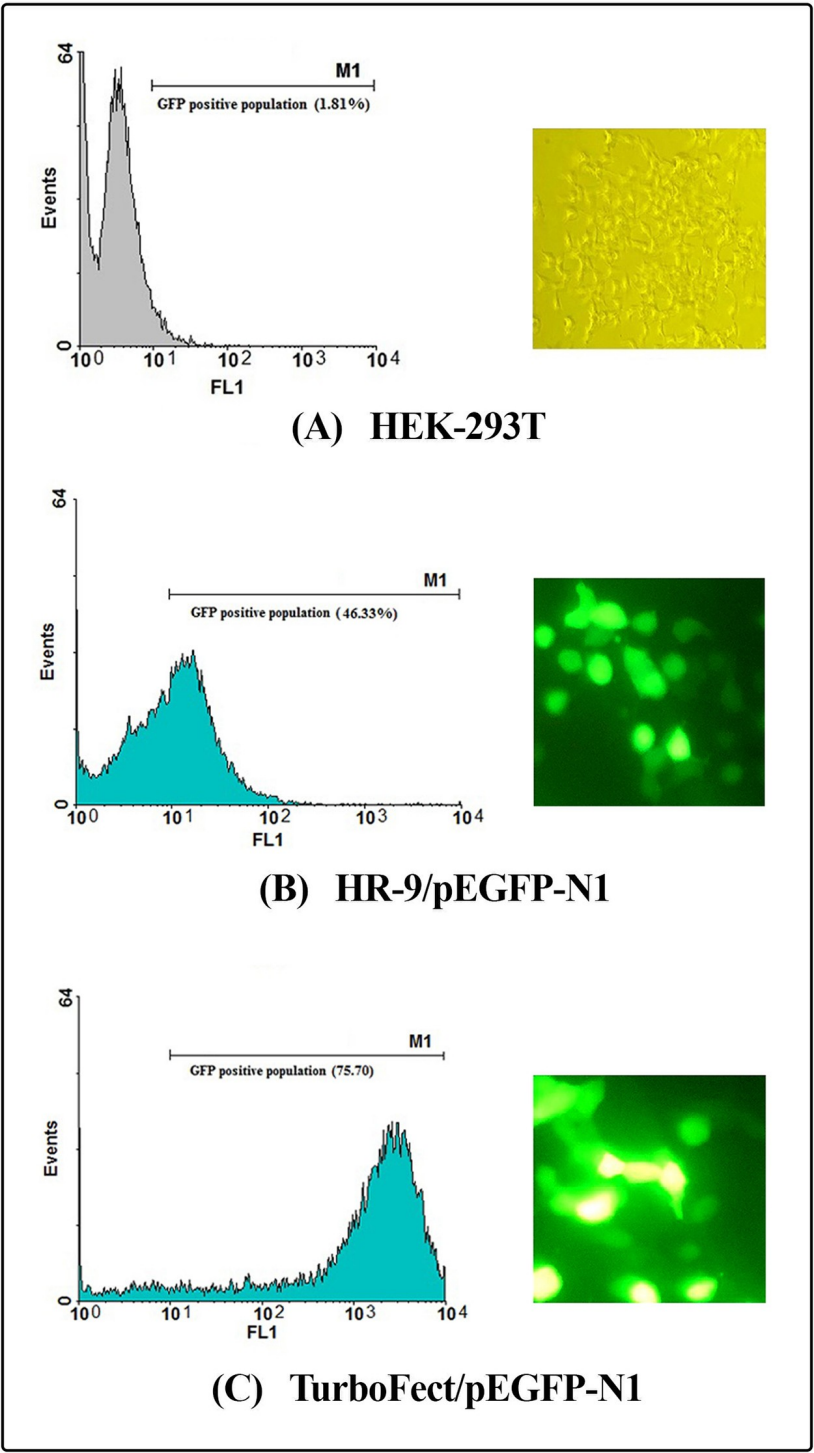
Transfection efficiency of the CPP/DNA complexes using flow cytometry and fluorescence microscopy. (A) HEK-293T as a negative control, (B) the HR9/pEGFP-N1 complexes, (C) the TurboFect/pEGFP-N1 complexes.

**Fig 9:**
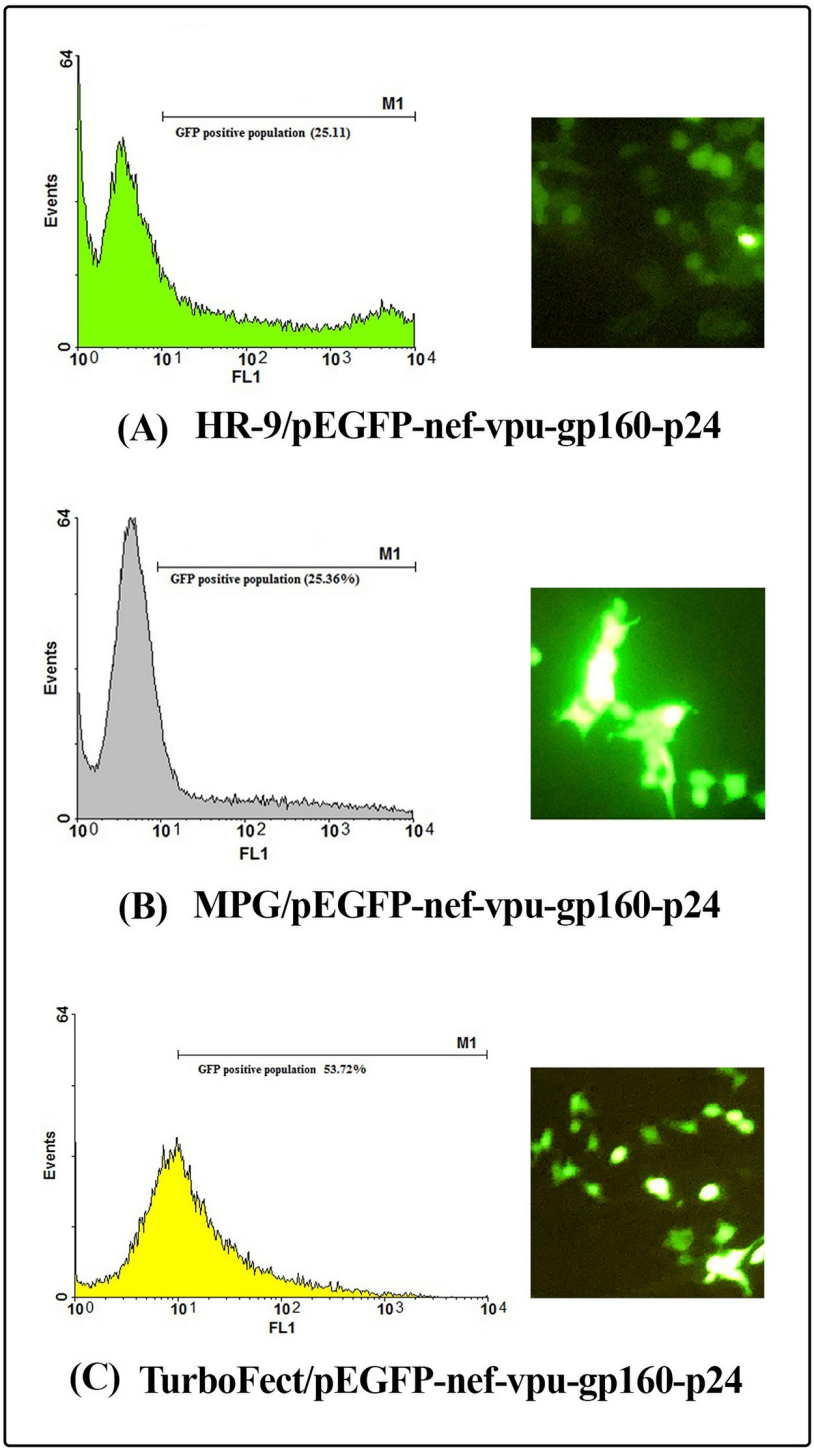
Transfection efficiency of the CPP/DNA complexes using flow cytometry and fluorescence microscopy. (A) the HR9/pEGFP-*nef-vpu-gp160-p24* complexes, (B) the MPG/pEGFP-*nef-vpu-gp160-p24* complexes, (C) the TurboFect/pEGFP-*nef-vpu-gp160-p24* complexes.

Internalization of the MPG/pEGFP-*nef-vif-gp160-p24*, MPG/pEGFP-*nef-vpu-gp160-p24*, HR-9/pEGFP-*nef-vif-gp160-p24* and HR9/pEGFP-*nef-vpu-gp160-p24* nanoparticles into HEK-293T cells was determined at N/P ratios of 10:1, 10:1, 5:1 and 5:1, respectively. According to the flow cytometry results, the percentage of GFP expressing cells was shown to be approximately 38.38 ± 1.34%, 25.36% ± 0.30, 54.95% ± 0.84, and 25.11% ± 0.36 for MPG/pEGFP-*nef-vif-gp160-p24*, MPG/pEGFP-*nef-vpu-gp160-p24*, HR9/pEGFP-*nef-vif-gp160-p24* and HR9/pEGFP-*nef-vpu-gp160-p24*, respectively. The transfection efficiency of pEGFP-*nef-vif-gp160-p24* and pEGFP-*nef-vpu-gp160-p24* delivered by TurboFect as a positive control was ~ 66.85% ± 0.81 and ~53.72% ± 0.15, respectively (Figs 8, 9, **and** 10). These results revealed that MPG and HR9 peptides could deliver effectively pEGFP-*nef-vif-gp160-p24* as compared to pEGFP-*nef-vpu-gp160-p24* suggesting the effect of Vif epitopes in cell penetration (*p* < 0.05; S2 Fig).

**Fig 10:**
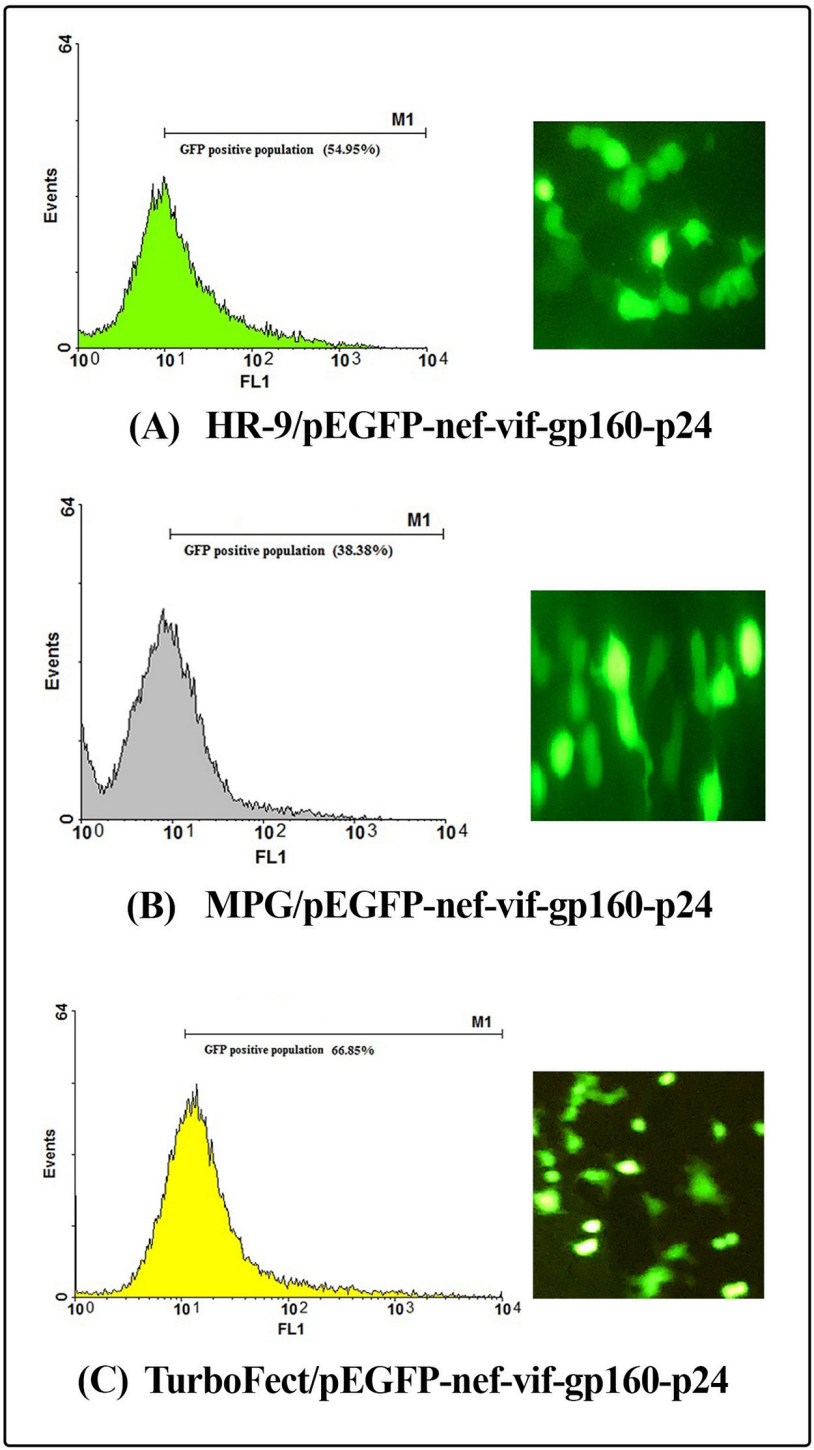
Transfection efficiency of the CPP/DNA complexes using flow cytometry and fluorescence microscopy. (A) the HR9/pEGFP-*nef-vif-gp160-p24* complexes, (B) the MPG/pEGFP-*nef-vif-gp160-p24* complexes, (C) the TurboFect/pEGFP-*nef-vif-gp160-p24* complexes.

#### The delivery of CyLoP-1 and LDP-NLS-mediated polyepitope peptides into the cells

**Fig 11:**
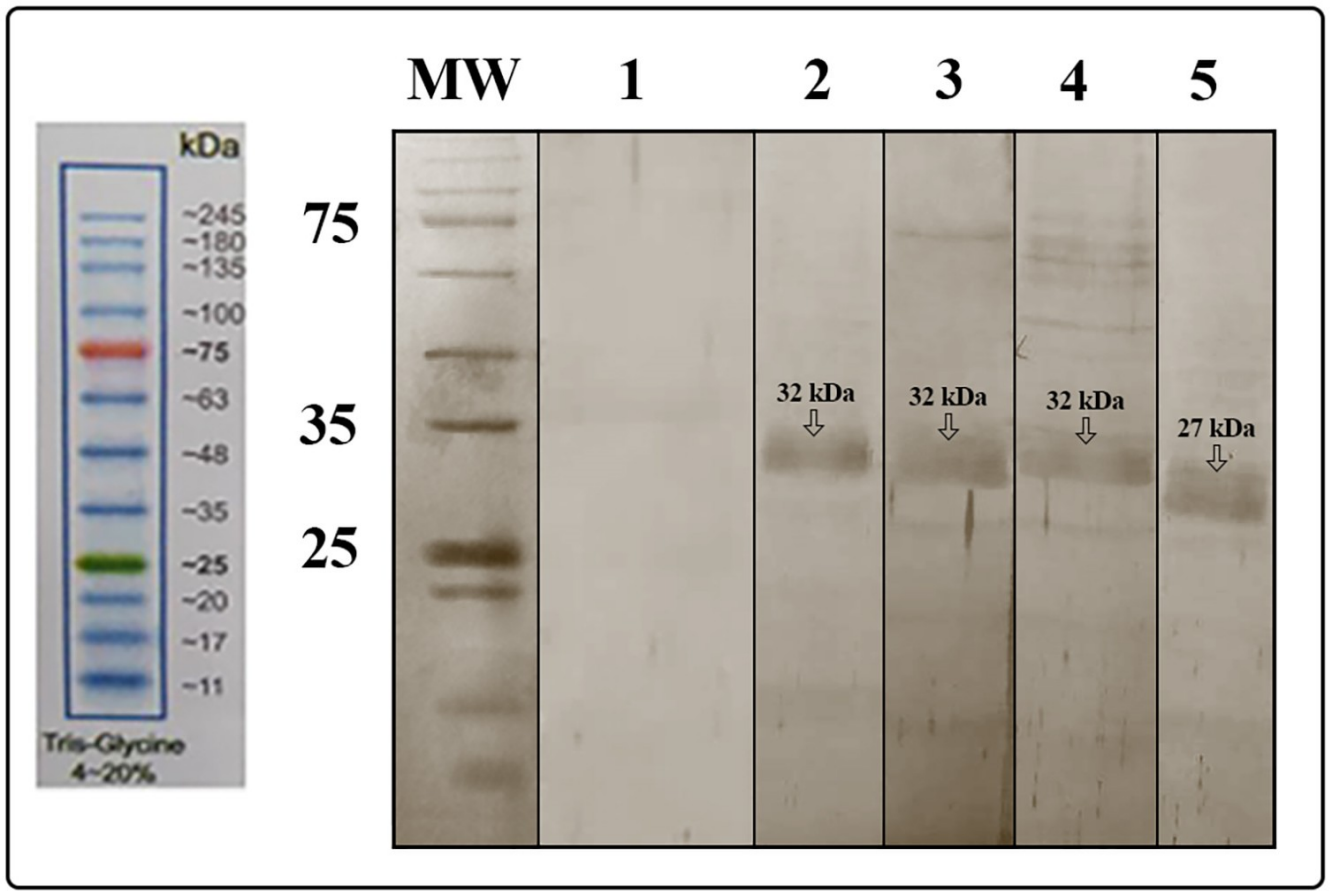
*In vitro* delivery of the CPP/peptide complexes in HEK-293T cells using western blot analysis. Lane 1: untransfected cells, Lane 2: transfected cells with TurboFect/ rNef-Vif-Gp160-P24, Lane 3: transfected cells with LDP-NLS/ rNef-Vif-Gp160-P24, Lane 4: transfected cells with CyLoP-1/rNef-Vif-Gp160-P24, Lane 5: transfected cells with green fluorescent protein (GFP)/TurboFect as positive control. A clear band of ~32 kDa was observed for delivery of rNef-Vif-Gp160-P24 into the cells. A clear band of ~ 27 kDa was observed for delivery of GFP by TurboFect as a positive control. Similar results were obtained for delivery of rNef-Vpu-Gp160-P24 by TurboFect, LDP-NLS and CyLoP-1 as a clear band of ~ 32 kDa (S3 Fig).

The delivery of the recombinant Nef-Vif-Gp160-P24 and Nef-Vpu-Gp160-P24 polyepitope peptides in HEK-293T cells was confirmed by western blot analysis. As shown in Fig 11, using anti-His antibody, a single band about 32 kDa was detected in the cells transfected with CyLoP-1 or LDP-NLS/rNef-Vif-Gp160-P24, and CyLoP-1 or LDP-NLS/rNef-Vpu-Gp160-P24 complexes compared to untransfected ones. These results revealed that CyLoP-1 and LDP-NLS could efficiently transfer rNef-Vif-Gp160-P24 and rNef-Vpu-Gp160-P24 polyepitope peptides into the cells.

#### *In vitro* cytotoxicity of the nanoparticles

MTT results indicated that the MPG/pEGFP*-nef-vif-gp160-p24*, MPG/pEGFP*-nef-vpu-gp160-p24*, HR9/pEGFP*-nef-vif-gp160-p24*, and HR9/pEGFP-*nef-vpu-gp160-p24* complexes at N/P ratios of 10:1, 10:1, 5:1 and 5:1 as well as the CyLoP-1/rNef-Vif-Gp160-P24, CyLoP-1/rNef-Vpu-Gp160-P24, LDP-NLS/rNef-Vif-Gp160-P24, and LDP-NLS/rNef-Vpu-Gp160-P24 complexes at molar ratio of 10:1 for all complexes were not cytotoxic in HEK 293T cell line. The percentage of cell viability for all complexes was between 94–97% and had no significant difference with untreated cells (97–99%; *p* > 0.05).

## Discussion

Over the past three decades, various studies have conducted to evaluate preventive and therapeutic vaccines against HIV-1. These studies have shown inefficient immune responses in the primary steps of development [33]. Thus, development of the recombinant DNA technology (rDNA) and bioinformatics tools are able to generate new vaccines against HIV-1 infections. The vaccines that were designated through rDNA approach were more efficient, safer and cheaper than traditional vaccines. To access these goals, further understanding of the virus genome especially determination of immunodominant epitopes for inducing the desired immune response is essential [34]. In several studies, it has been shown that the multi-epitope-based vaccines against HIV-1 were successful in preclinical trials. For example, Letourneau *et al*. designed and tested a vaccine harboring 14 conserved epitopes of the HIV-1 in BALB/c mice. The results indicated the stimulation of T cell responses and subsequently secretion of IFN-γ, IL-2 and TNF-α cytokines [35]. In another study, Wilson *et al*. showed the potency of a therapeutic epitope-based HIV-1 DNA vaccine encoding 21 different epitopes derived from structural and regulator proteins in a phase I clinical trial for HIV-1-infected subjects receiving highly active antiretroviral therapy. However, the cytotoxic T cell (CTL) responses were low [36], thus it was required to improve immune responses using delivery systems. Recent studies on multi-epitope vaccines have indicated an essential improvement of their efficacy [37–42]. In current study, the multi-epitope DNA and peptide constructs harboring the conserved immunodominant T-cell epitopes from several antigens were designed. Rowland-Jones *et al*. broadly studied the conserved peptide epitopes for T-cell recognition [43]. It was proven that an efficient vaccine against HIV-1 should enhance the long-term cellular immune responses [13]. In this line, immune-bioinformatics methods were used to select the highly conserved epitopes and predict T helper epitopes [44]. In this study, highly conserved epitopes from HIV-1 structural and accessory proteins (*i*.*e*., Nef, Vif, Vpu, Gp160 & P24) were chosen. The efficiency of these proteins as promising candidates for vaccine development against HIV-1 has been proved in various studies [43, 45–51]. Tarosso *et al*. showed that the constructs harboring *nef* and *vif* epitopes predicted by NetMHC induced broader cellular immune responses in infected subjects [52]. Herein, we designed two multiepitope-based constructs based on binding affinity, identification scores and interaction similarity scores in molecular docking. These constructs were different in harboring Vif- and Vpu-derived epitopes. It was observed that the most regions of P24 were highly conserved and immunogenic, thus we preferred to use p24_8−151_ instead of individual epitopes. Moreover, we developed the multiepitope constructs containing a set of overlapping peptide epitopes (7–24 residues in length). In fact, long peptides could stimulate more potent and higher immune responses than short peptides. In several studies, it has proved that vaccination with a set of long overlapping peptide epitopes successfully elicited immune responses against HPV16 E6 and E7 in individuals with cervical cancer, and also against Epstein-Barr virus (EBV) [2, 53]. Uptake of overlapping epitopes by dendritic cells (DCs) could induce strong and potent cellular immune responses (CD4^+^ helper T cells and CD8^+^ T cells) [53]. As known, native conformation is necessary for induction of humoral immunity, but conformation is not essential for eliciting cellular immunity [54].

On the other hand, it was observed that the poor cellular permeability for large and hydrophilic molecules limited the therapeutic use of peptides and proteins. CPPs were able to efficiently transfer biological cargoes including nucleotides, peptides, proteins and other nanoparticles across the plasma membrane. Compared to lipid- and polymer-based delivery systems, they are non-toxic, high stable and cost-effective carriers with low immunogenicity [55]. In current study, MPG and HR9 CPPs were used as DNA delivery systems, and LDP-NLS and CyLoP-1 CPPs were applied to deliver polyepitope peptides *in vitro*. The pEGFP-*nef-vif-gp160-p24* and pEGFP-*nef-vpu-gp160-p24* were prepared for *in vitro* gene delivery. Furthermore, the His-tagged recombinant Nef-Vif-Gp160-P24 and Nef-Vpu-Gp160-P24 polyepitope peptides were expressed in *E*. *coli Rossetta* and purified by affinity chromatography using Ni-NTA column. The researchers showed that MPG, a 27 residue chimeric peptide, was able to non-covalently bind to single strand (ss)- and double strand (ds)-oligonucleotides to form stable complexes. The nucleic acids were protected from degradation by DNase in these complexes [56]. Regarding the reports, MPG CPP could be utilized as an efficient carrier for siRNA or plasmid DNA *in vitro* or *in vivo* [31, 57]. HR9 CPP (Histidine-rich nonaarginine) was utilized effectively to deliver DNA into the living cells, as well [58]. On the other hand, LDP (Latarcin-derived peptide) conjugated with SV40 NLS could deliver proteins into HeLa cells. Moreover, a significant reduction in cell cytotoxicity was reported for LDP-NLS as compared to LDP [27]. CyLoP-1 CPP was also capable of penetrating into mammalian cell lines as well as plant cells without inducing notable toxicity in HeLa cells and wheat protoplasts [26, 59]. Our findings indicated that MPG and HR9 CPPs at N/P ratios of 10:1 and 5:1, and LDP-NLS and CyLoP-1 CPPs at molar ratios of 10:1 (CPP: peptide) could non-covalently bind to DNA, and peptides to form stable nanoparticles, respectively. Our data were consistent with previous studies which the optimal complex formation for successful delivery of DNA constructs was achieved at molar ratios of 10:1 and 5:1 for MPG/cargo and HR9/cargo, respectively [20, 31, 60]. Our results confirmed a significant reduction in size of the CPP/DNA or CPP/peptide nanoparticles as compared to DNA and peptides, alone. Moreover, Zetasizer analysis of the CPP/DNA or CPP/peptide nanoparticles showed positive charges in comparison with negatively charged DNA or peptides. According to previously published data, cationic particles were effectively uptaken by DCs and macrophages resulting in strong immune responses [61]. As observed in our previous study, the effective immune responses and protection were generated by MPG/HPV16 E7 DNA nanoparticles with a positive surface charge. Indeed, MPG could non-covalently bind to HPV16 E7 DNA at N/P ratio of 10:1 and form stable nanoparticles (∼110–130 nm). This study is consistent with our results indicating high affinity of MPG for transferring DNA constructs into mammalian cells at a molar ratio of 10:1 [31].

Herein, *in vitro* delivery of the MPG/DNA, HR9/DNA, LDP-NLS/peptide, and CyLoP-1/peptide complexes was confirmed by flow cytometry or western blotting. One clear band of ~32 kDa related to Nef-Vpu-Gp160-P24 and Nef-Vif-Gp160-P24 was observed in the transfected cells with the CPP/peptide nanoparticles using anti-His antibody. The flow cytometry results indicated that delivery of the HR9/pEGFP-*nef-vif-gp160-p24*, HR9/pEGFP-*nef-vpu-gp160-p24*, MPG/pEGFP-*nef-vif-gp160-p24*, and MPG/pEGFP-*nef-vpu-gp160-p24* complexes was confirmed in approximately 54.95%, 25.11%, 38.38%, and 25.36% of the transfected cells, respectively, indicating that HR9 and MPG were able to effectively deliver DNA constructs. Liu *et al* showed that HR9 peptide could successfully translocate plasmid DNA, semiconductor quantum dots (QDs) and red fluorescent proteins (RFPs) across cell membrane [58, 62]. For insect transgenesis, arginine-rich CPPs such as HR9 were also shown to be a non-toxic and functional tool [63]. Our previous study also indicated that HR9 CPP at N/P ratio of 5:1 could deliver pEGFP-NS3 and pEGFP-Hsp27-NS3 across HEK-293 T cells (20.51% ± 0.94 and 33.34% ± 0.94, respectively) (60). Our results revealed that the efficiency of both MPG and HR9 peptides was significantly higher for pEGFP-*nef-vif-gp160-p24* than for pEGFP-*nef-vpu-gp160-p24* into HEK-293T cells suggesting higher penetration of Vif as compared to Vpu. Thus, it may be considered as an antigen carrier. Compared to untreated cells, the MTT assay showed no significant toxicity in HEK-293T cells treated with different ratios of the nanoparticles, similar to other published reports [20, 60, 64]. However, reduction of the dose of constructs complexed with CPPs was important to stimulate immune responses in vaccine development [65, 66].

There are other *in vitro*/ *in vivo* studies on using CPPs in vaccine development. For example, the immunostimulatory properties of HIV-1 Nef DNA and protein constructs were evaluated using four CPPs (HR9, MPG, M918, and penetratin) as a gene or protein carrier in BALB/c mice. The data indicated that the simultaneous use of M918 and MPG CPPs as protein and gene carriers could improve HIV-1 Nef-specific B- and T-cell immune responses as a promising approach for development of HIV-1 monovalent vaccine [67]. It was observed that M918 CPP could increase the penetration of HIV-1 Nef protein into the cells [68]. Another study showed highly efficient CPPs (P28 and MPG) for the controlled delivery of HPV16 E7 antigen, *in vitro and in vivo*. It was shown that the groups vaccinated with rE7+ P28/rE7+ P28, MPG+ E7 DNA/P28+ rE7, and E7 DNA+ MPG/E7 DNA+ MPG nanovaccines displayed complete protection and remained tumor-free > 60 days after treatment. Thus, P28 and MPG CPPs were utilized to develop HPV therapeutic vaccines as promising protein and gene delivery systems, respectively [64]. On the other hand, the hPP10 CPP produced the best transfection result for HPV E7 protein in HEK-293T cells (~ 63.66%) compared to TurboFect commercial reagent (~ 32.95%) [69]. Furthermore, the use of Tat (PTD)-Nef antigen in prime-boost strategy along with Cady-2 CPP significantly enhanced the Nef-specific T cell responses [70].

In conclusion, we designed two different multiepitope constructs against HIV-1 infection. In order to clarify the effect of epitopes, two constructs with only one variable factor were compared. In one construct, Vif epitopes were used and in another one, Vpu epitopes were replaced. In equal conditions, the nanoparticles harboring Vif epitopes could be transferred into cells more than the nanoparticles harboring Vpu epitopes. Furthermore, cell penetrating peptides were effective for both DNA and peptide delivery with low toxicity *in vitro*. However, further studies are required to determine the immunological and protective effects of the designed constructs *in vivo*.

## Supporting information

S1 Table T cell epitope prediction of MHC-I and MHC-II with their scores. (TIF)

S1 Fig The stability of HR9/DNA and MPG/DNA nanoparticles against DNA alone in the presence of serum. DNA is pEGFP-*nef-vif-gp160-p24* and /or pEGFP-*nef-vpu-gp160-p24*.
(TIF)

S2 Fig Results of flow cytometry. Column 1: MPG/pEGFP-*nef-vif-gp160-p24*, Column 2: MPG/pEGFP-*nef-vpu-gp160-p24*, Column 3: HR9/pEGFP-*nef-vif-gp160-p24*, Column 4: HR-9/pEGFP-*nef-vpu-gp160-p24*, Column 5: TurboFect/pEGFP-*nef-vif-gp160-p24*, Column 6: TurboFect/pEGFP-*nef-vpu-gp160-p24*. * *p* < 0.05; ** *p* < 0.01.
(TIF)

S3 Fig *In vitro* delivery of the CPP/peptide complexes in HEK-293T cells using western blot analysis. Lane 1: untransfected cells, Lane 2: transfected cells with TurboFect/ rNef-Vpu-Gp160-P24, Lane 3: transfected cells with LDP-NLS/ rNef-Vpu-Gp160-P24, Lane 4: transfected cells with CyLoP-1/rNef-Vpu-Gp160-P24.
(TIF)

S1 Raw Fig Expression and purification of Nef-Vif-Gp160-P24 protein (for Fig 6A). (TIF)

S2 Raw Fig Expression and purification of Nef-Vpu-Gp160-P24 protein (for Fig 6B). (TIF)

S3 Raw Fig *In vitro* delivery of the CPP/peptide complexes in HEK-293 T cells using western blot analysis (for Fig 11 and S3 Fig). (TIF)
