# Supplementary figures and images for: Comparison of HIV-1 Vif and Vpu accessory proteins for delivery of polyepitope constructs harboring Nef, Gp160 and P24 using various cell penetrating peptides
Source: PLoS One. 2019 Oct 31;14(10):e0223844. doi: 10.1371/journal.pone.0223844 (PMC6822742; doi:10.1371/journal.pone.0223844)

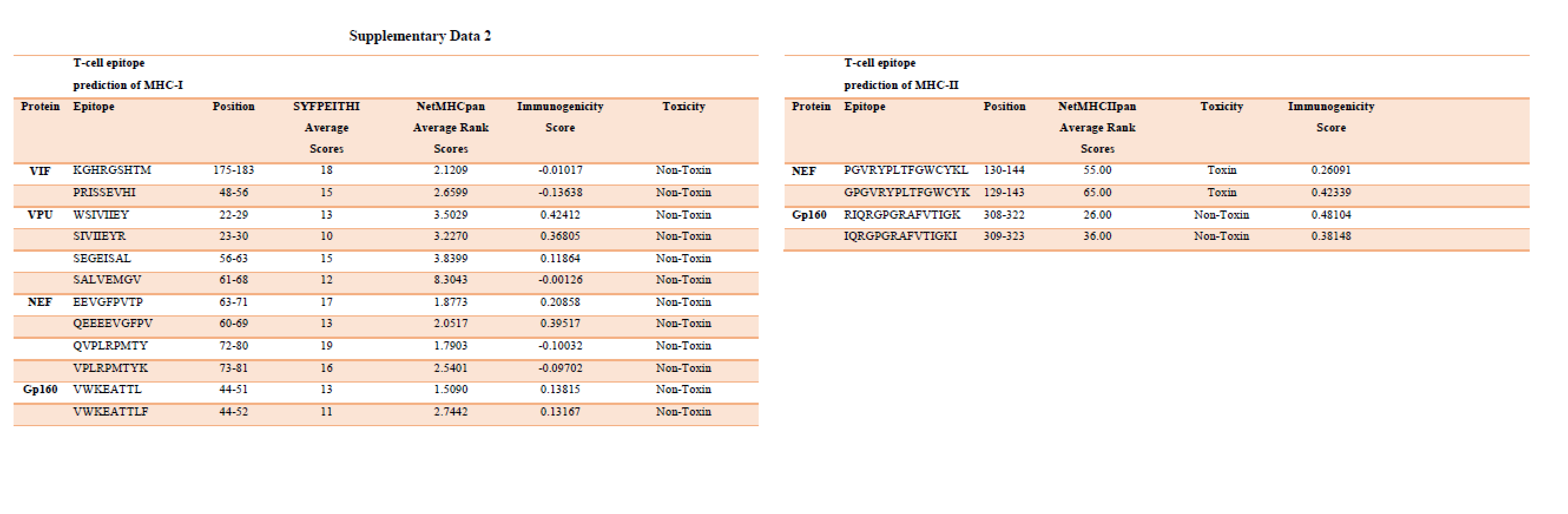

Supplement: S1 Table — (TIF) [file pone.0223844.s001.tif]

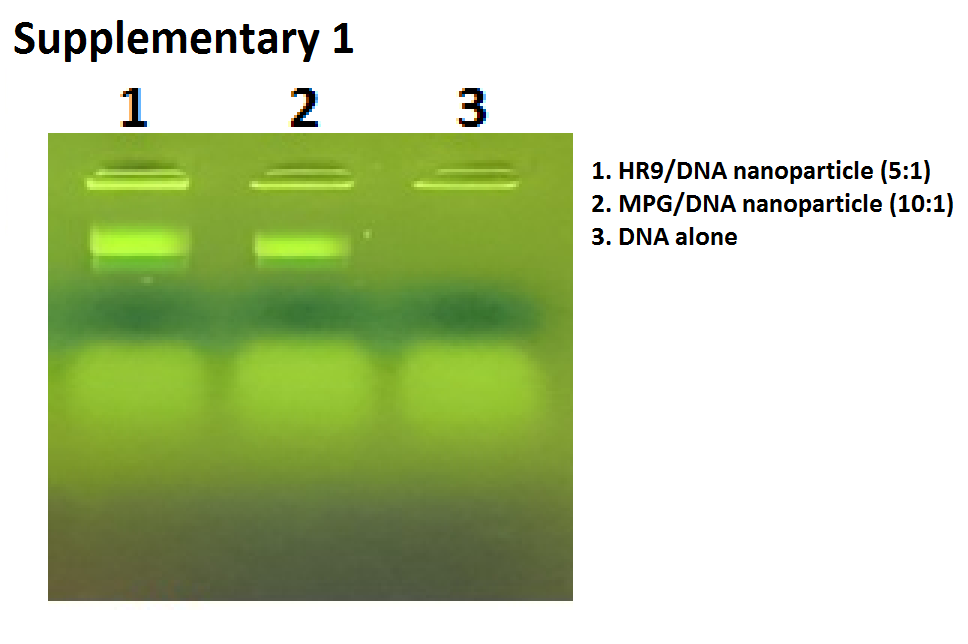

Supplement: S1 Fig — DNA is pEGFP-nef-vif-gp160-p24 and /or pEGFP-nef-vpu-gp160-p24. (TIF) [file pone.0223844.s002.tif]

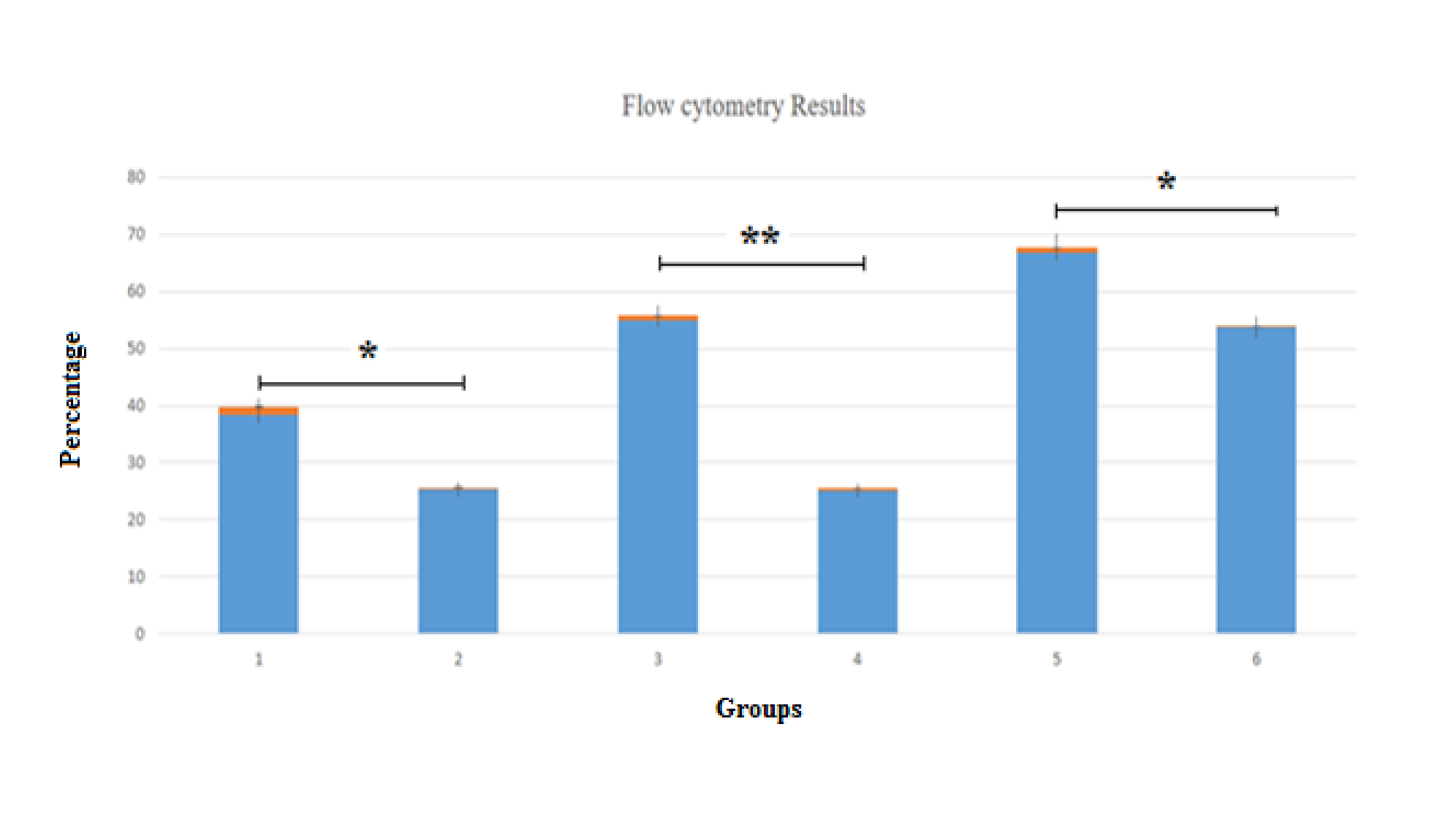

Supplement: S2 Fig — Column 1: MPG/pEGFP-nef-vif-gp160-p24, Column 2: MPG/pEGFP-nef-vpu-gp160-p24, Column 3: HR9/pEGFP-nef-vif-gp160-p24, Column 4: HR-9/pEGFP-nef-vpu-gp160-p24, Column 5: TurboFect/pEGFP-nef-vif-gp160-p24, Column 6: TurboFect/pEGFP-nef-vpu-gp160-p24. * p < 0.05; ** p < 0.01. (TIF) [file pone.0223844.s003.tif]

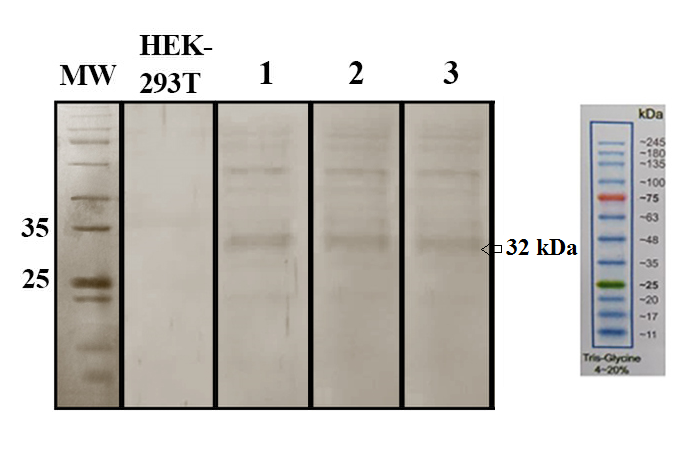

Supplement: S3 Fig — Lane 1: untransfected cells, Lane 2: transfected cells with TurboFect/ rNef-Vpu-Gp160-P24, Lane 3: transfected cells with LDP-NLS/ rNef-Vpu-Gp160-P24, Lane 4: transfected cells with CyLoP-1/rNef-Vpu-Gp160-P24. (TIF) [file pone.0223844.s004.tif]

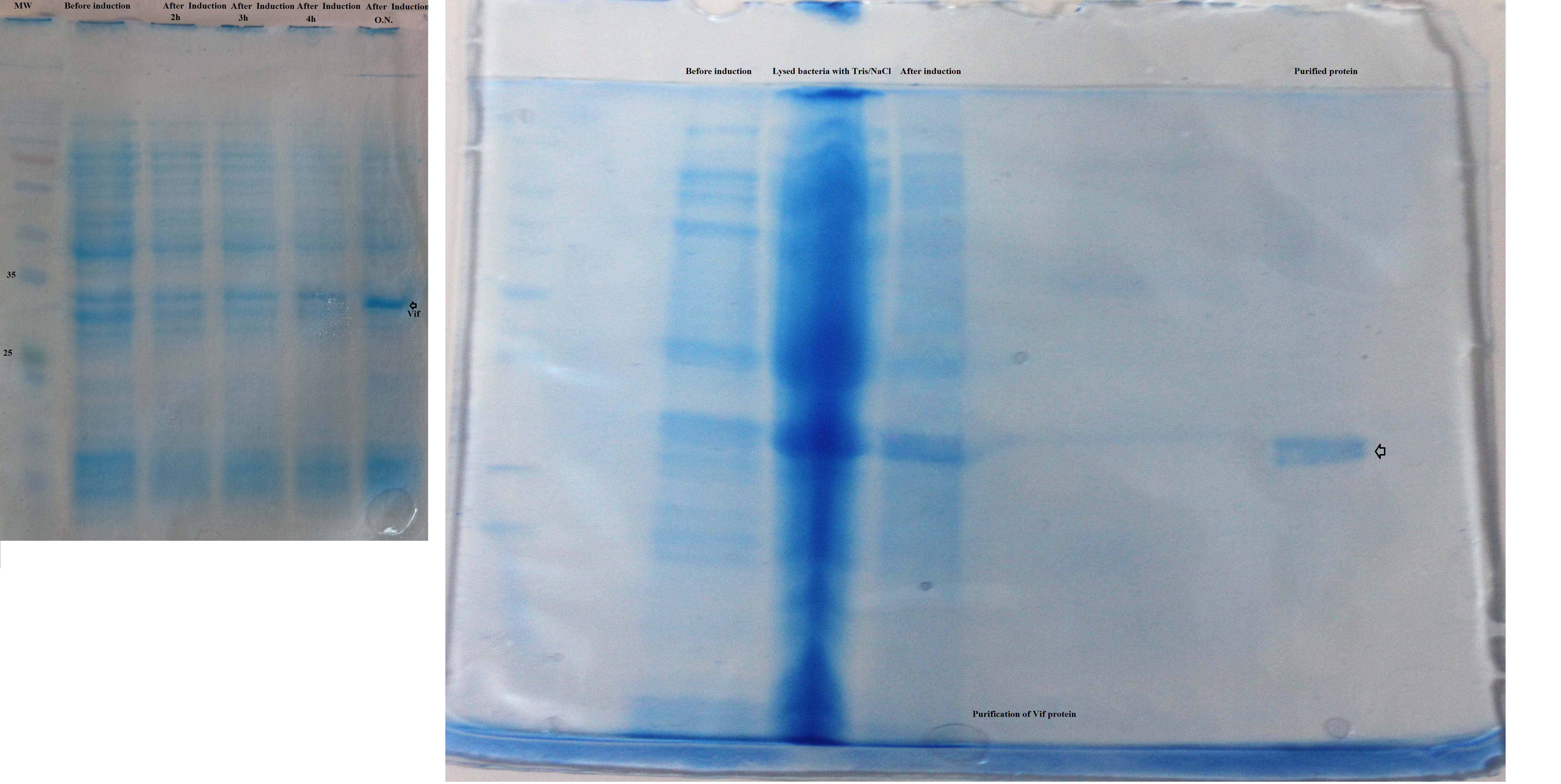

Supplement: S1 Raw Fig — (TIF) [file pone.0223844.s005.tif]

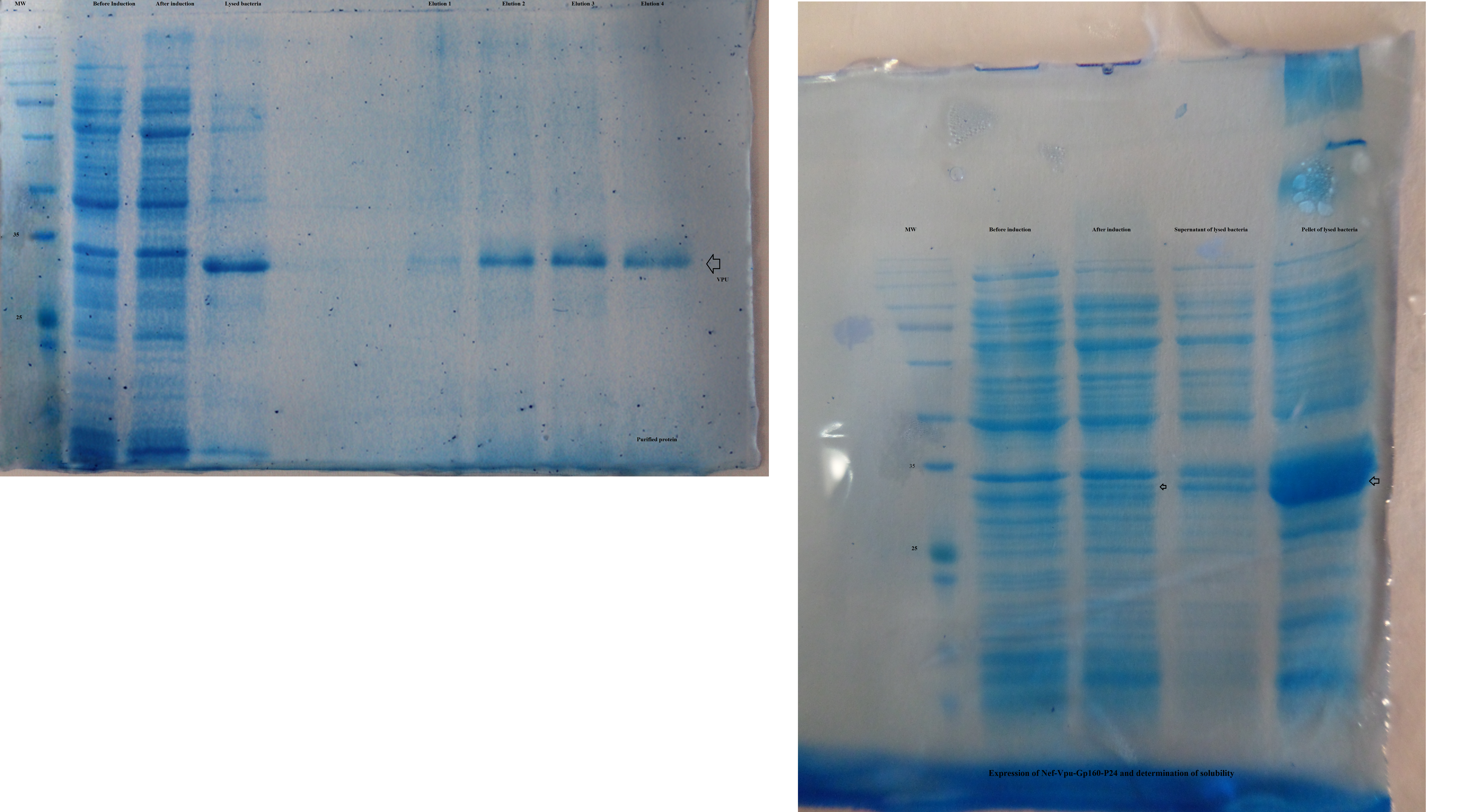

Supplement: S2 Raw Fig — (TIF) [file pone.0223844.s006.tif]

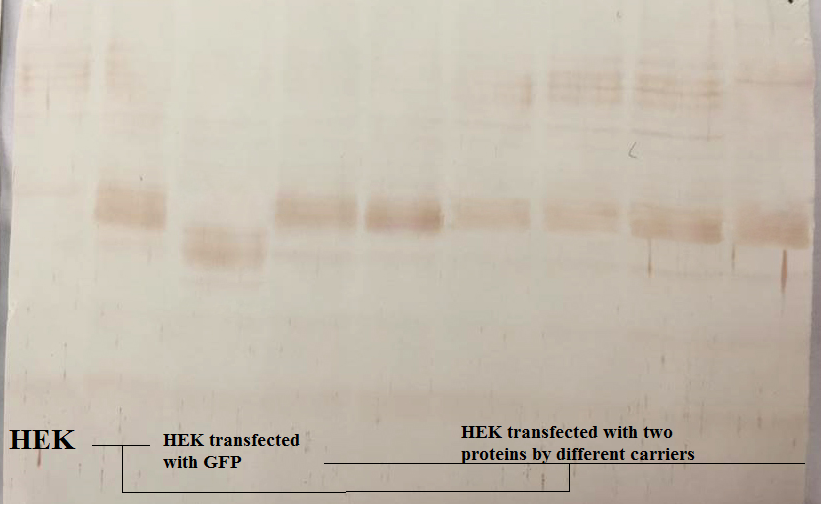

Supplement: S3 Raw Fig — (TIF) [file pone.0223844.s007.tif]
